# Decreased TMPRSS2 expression by SARS-CoV-2 predicts the poor prognosis of lung cancer patients through metabolic pathways and immune infiltration

**DOI:** 10.18632/aging.203823

**Published:** 2022-01-11

**Authors:** Xiaopeng Liu, Bing Liu, Yanan Shang, Pengxiu Cao, Jiajie Hou, Fei Chen, Bo Zhang, Yumei Fan, Ke Tan

**Affiliations:** 1 Ministry of Education Key Laboratory of Molecular and Cellular Biology, Key Laboratory of Animal Physiology, Biochemistry and Molecular Biology of Hebei Province, College of Life Sciences, Hebei Normal University, Shijiazhuang, Hebei 050024, China; 2 Department of Neurosurgery, The Second Hospital of Hebei Medical University, Shijiazhuang, Hebei 050000, China

**Keywords:** TMPRSS2, SARS-CoV-2, lung cancer, prognostic biomarker, immune infiltration, COVID-19

## Abstract

Severe acute respiratory syndrome coronavirus 2 (SARS-CoV-2) has rapidly spread around the world and became a global pandemic in 2020. One promising drug target for SARS-CoV-2 is the transmembrane protease serine 2 (TMPRSS2). This study was designed to explore the expression status, prognostic significance and molecular functions of TMPRSS2 in lung cancer. TMPRSS2 expression was investigated using the TIMER, Oncomine, UALCAN, GEO, HPA and TCGA databases. The prognostic value of TMPRSS2 was examined using Cox regression and a nomogram. KEGG, GO and GSEA were performed to investigate the cellular function of TMPRSS2 in lung cancer. The relationship between TMPRSS2 and immune infiltration was determined using the TIMER and CIBERSORT algorithms. TMPRSS2 mRNA and protein expression was significantly reduced in lung cancer. Decreased TMPRSS2 expression and increased DNA methylation of TMPRSS2 were associated with various clinicopathological parameters in patients with lung cancer. Low TMPRSS2 mRNA expression also correlated with poor outcome in lung cancer patients. Moreover, a nomogram was constructed and exhibited good predictive power for the overall survival of lung cancer patients. KEGG and GO analyses and GSEA implied that multiple immune- and metabolism-related pathways were significantly linked with TMPRSS2 expression. Intriguingly, TMPRSS2 expression associated with immune cell infiltration in lung cancer. More importantly, TMPRSS2 expression was markedly decreased in SARS-CoV-infected cells. These findings indicate that TMPRSS2 may be a promising prognostic biomarker and therapeutic target for lung cancer through metabolic pathways and immune cell infiltration.

## INTRODUCTION

COVID-19 (coronavirus disease 2019) has emerged from infection with SARS-CoV-2 (severe acute respiratory syndrome coronavirus 2) and has created a global epidemic with over 257 million patients in most countries of the world and more than 5.1 million deaths (updated on 24 November 2021) [1–3]. Recently studies have demonstrated that both transmembrane protease serine 2 (TMPRSS2) and angiotensin I converting enzyme 2 (ACE2) are crucial for the entry of SARS-CoV-2 into host cells [4–6]. Both TMPRSS2 and ACE2 are expressed in lung tissues, as implicated in the clinical manifestations of COVID-19 [4–6]. TMPRSS2 is also expressed in other tissues, such as the prostate epithelium, cardiac endothelium, digestive tract and kidney, indicating that these organs may be the most susceptible targets for SARS-CoV-2 infection [7, 8]. Consistent with these observations, SARS-CoV-2 infection can result in multisystemic, life-threatening complications. Similar to SARS and MERS-CoV, SARS-CoV-2 mainly affects the function of the lower respiratory tract [9, 10]. In more severe cases, it can induce acute respiratory distress syndrome and severe lung damage, leading to inflammation and pulmonary vasculopathy [2, 9].

Lung cancer is the most frequent malignancy and the leading cause of cancer-related death worldwide [10]. NSCLC (Non-small cell lung cancer) is the most common pathological type of lung cancer and is responsible for 85% of all lung cancers [10, 11]. Radiotherapy, chemotherapy, surgical resection, and immunotherapy are common therapies employed to treat lung cancer [10, 11]. Due to problems in early diagnosis, patients with NSCLC are often diagnosed at advanced stages [10, 11]. Patients with lung cancer are more vulnerable to various infections due to poor healthy condition, accompanying chronic diseases, and immunosuppression induced by tumor and/or antitumor therapies. Therefore, cancer patients who are infected by SARS-CoV-2 may suffer worse outcomes than other individuals [12]. Indeed, previous studies have demonstrated that cancer patients with coronavirus infections may be more susceptible to higher morbidity and mortality rates. In a study at a hospital in Wuhan, China, cancer patients accounted for 1% of the total prevalence of COVID-19, which is substantially higher than the 0.29% of the total incidence of cancer in the Chinese population [13, 14]. Lung cancer patients seem to be more susceptible to SARS-CoV-2 infection [12, 15]. Therefore, the association between immune infiltration in cancer patients and the susceptibility or severity of COVID-19 needs to be fully elucidated.

Previous studies have revealed that multiple viruses, such as influenza virus, Ebola virus, MERS-CoV, and SARS-CoV, use host cell proteases to facilitate the activation of their envelope glycoproteins [16]. The cleavage and activation of the spike protein (S protein) of SARS-CoV are regulated by TMPRSS2 [5, 6]. TMPRSS2 is a protease that belongs to the type II transmembrane serine protease family and is required to activate S protein to cause virus-cell membrane fusion and promote coronaviruses to inter into the host cell [5, 6]. Several animal models have demonstrated that TMPRSS2-KO (TMPRSS2-knockout) mice can be protected from severe pathology and death after influenza virus infection [16–19]. Knockout of TMPRSS2 prevents the spread of MERS-CoV and SARS-CoV in the airway of a mouse model by alleviating inflammatory cytokine production [17, 18]. The reduced TMPRSS2 expression alters the primary site of infection and the transmission of the virus in the airway, leading to less severe immunopathology [17–19]. In contrast, overexpression of certain TMPRSS2 variants in animals results in an increased risk of severe outcomes after infection with A (H1N1) pmd09 influenza [20].

Given that TMPRSS2 is a promising drug target for SARS-CoV-2, this study aimed to investigate the expression profile, determine the prognostic potential of TMPRSS2, and estimate the association between TMPRSS2 and immune cell infiltration in lung cancer. We observed that TMPRSS2 expression was decreased in lung cancer tissues compared with adjacent nontumor tissues. TMPRSS2 expression was reduced in different tumor stages and linked with lymph node metastasis. Subsequently, TMPRSS2 expression was negatively and significantly related with the prognosis of lung cancer patients. Kyoto Encyclopedia of Genes and Genomes (KEGG) and gene Ontology (GO) analyses and gene set enrichment analysis (GSEA) demonstrated that various metabolic and immune-related pathways were strongly associated with TMPRSS2 expression. Moreover, there was a significant correlation between TMPRSS2 expression and the infiltration abundances of CD8+ T cells, CD4+ T cells, B cells, neutrophils, macrophages, and dendritic cells in lung cancer. Importantly, TMPRSS2 expression was significantly decreased during SARS-CoV-2 infection. These findings emphasize a notable role of TMPRSS2 in carcinogenesis.

## RESULTS

### TMPRSS2 expression across cancers

**Figure 1:**
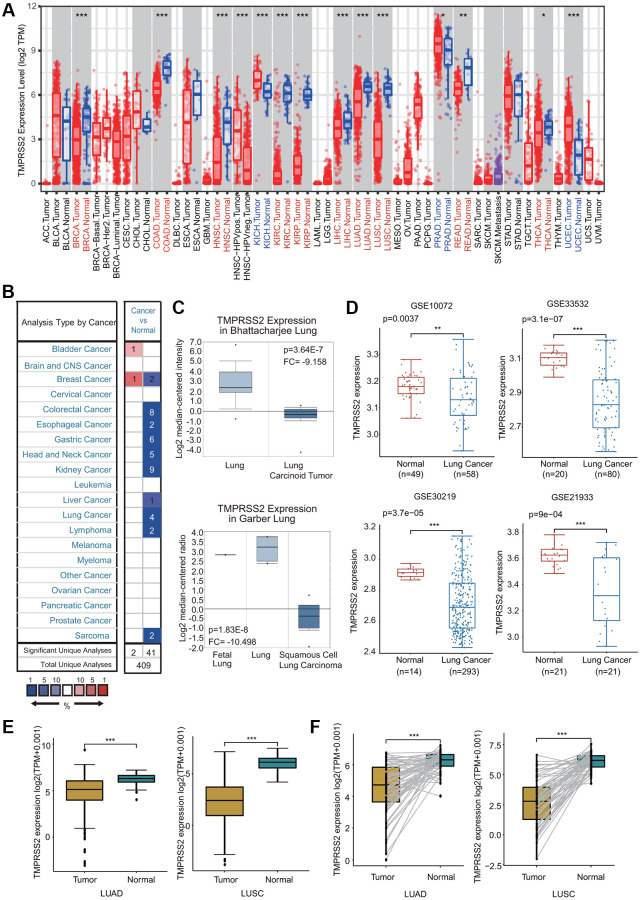
**TMPRSS2 expression in lung cancer.** (**A**) The mRNA expression of TMPRSS2 in different cancers from the TIMER database. (**B**) Upregulated or downregulated expression of TMPRSS2 in various tumors compared with normal tissues in the Oncomine database. (**C**) Box plots showing TMPRSS2 mRNA level in different types of lung cancer patients and normal individuals from the Oncomine database. (**D**) TMPRSS2 mRNA level in lung cancer patients and normal individuals in the GSE10072 (normal, *n* = 49; lung cancer, *n* = 58), GSE33532 (normal, *n* = 20; lung cancer, *n* = 80), GSE30219 (normal, *n* = 14; lung cancer, *n* = 293) and GSE21933 (normal, *n* = 21; lung cancer, *n* = 81) datasets. (**E**) TMPRSS2 expression is decreased in lung cancer (*n* = 81) compared with noncancerous adjacent tissues (*n* = 81) from the TCGA database. (**F**) TMPRSS2 expression in 58 and 50 matched LUAD and LUSC samples and adjacent normal lung tissues in the TCGA database was determined. ^*^p < 0.05, ^**^p < 0.01, ^***^p < 0.001.

We first estimated TMPRSS2 expression at the gene transcription level in various human tissues and organs using the GTEx database. Consistent with previous studies, we observed that TMPRSS2 was highly expressed in internal tissues (small intestine, kidney, colon, lung, liver, esophagus, stomach and bladder), secretory tissues (pancreas, thyroid, breast, salivary gland, pituitary and skin) and reproductive tissues (prostate and testis) (Supplementary Figure 1A). We then investigated TMPRSS2 expression in common tumors and their adjacent normal tissues through the TIMER database. The TMPRSS2 mRNA levels in BRCA, COAD, KIRC, KIRP, HNSC, LUAD, LUSC, LIHC, READ and THCA were significantly reduced compared with those in corresponding adjacent normal tissues (Figure 1A). In contrast, a significant increase in TMPRSS2 expression was observed in KICH, PRAD, and UCEC (Figure 1A). Moreover, TMPRSS2 mRNA expression levels in different cancer types were assessed through the Oncomine database. In 41 of the 43 unique analyses, TMPRSS2 expression was downregulated (Figure 1B). TMPRSS2 was significantly decreased in LUSC, LUAD, lung carcinoid tumor, small cell lung carcinoma, and large cell lung carcinoma (Figure 1C; Supplementary Figure 1B). Consistently, lower TMPRSS2 mRNA expression was found in four GEO cohorts, GSE10072, GSE33532, GSE30219 and GSE21933 (Figure 1D). Moreover, we compared TMPRSS2 expression in lung cancer using the TCGA dataset, and the results demonstrated that TMPRSS2 expression was significantly downregulated in lung cancer tissues (Figure 1E). TMPRSS2 expression in 58 and 50 paired LUAD and LUSC samples and corresponding adjacent normal samples was analyzed, and our results suggested a marked decrease in TMPRSS2 in lung cancer (Figure 1F). Additionally, we assessed TMPRSS2 expression in multiple cancer cell lines based on the CCLE database and found that TMPRSS2 expression was high in COAD, BRCA, PAAD, STAD and PRAD cells but low in AML, MESO, ALL and LUSC cells (Supplementary Figure 1C).

### Correlation between TMPRSS2 expression and clinicopathological characteristics

**Figure 2:**
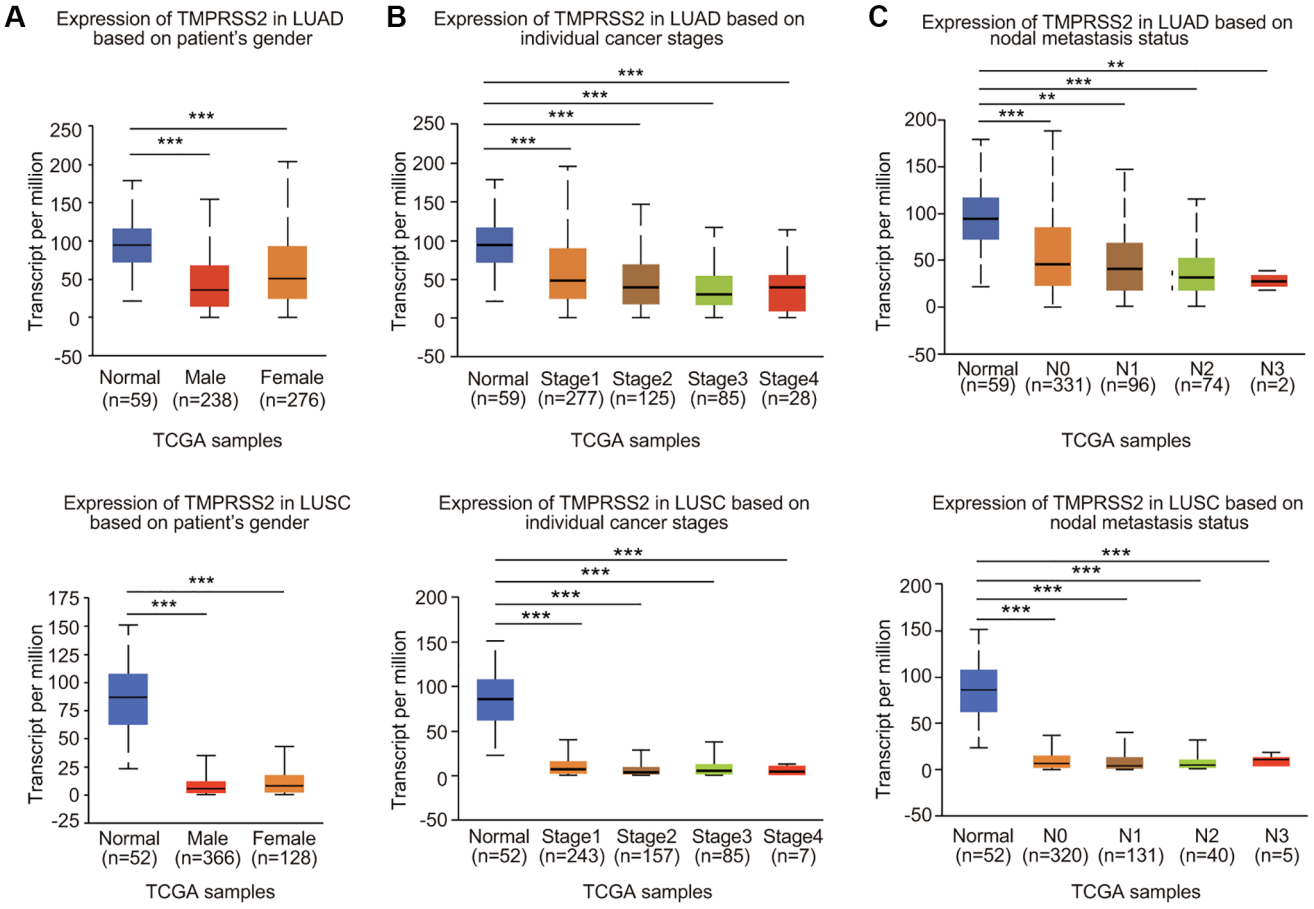
**Relationship between TMPRSS2 expression and clinicopathological parameters of lung cancer patients.** TMPRSS2 expression was assessed in (**A**) male and female LUAD (normal, *n* = 59; male, *n* = 238; female, *n* = 276) and LUSC (normal, *n* = 52; male, *n* = 366; female, *n* = 128) patients, (**B**) patients with different stages of LUAD (normal, *n* = 59; stage 1, *n* = 277; stage 2, *n* = 125, stage 3, *n* = 85; stage 4, *n* = 28) and LUSC (normal, *n* = 52; stage 1, *n* = 243; stage 2, *n* = 157, stage 3, *n* = 85; stage 4, *n* = 7), (**C**) patients with different nodal metastasis statuses of LUAD (normal, *n* = 59; N0, *n* = 331; N1, *n* = 96, N2, *n* = 74; N3, *n* = 2) and LUSC (normal, *n* = 52; N0, *n* = 320; N1, *n* = 131, N2, *n* = 40; N3, *n* = 5). ^*^p < 0.05, ^**^p < 0.01, ^***^p < 0.001.

We then examined the expression profiles of TMPRSS2 in lung cancer based on clinicopathological characteristics. Analysis mining of the UALCAN database revealed that TMPRSS2 expression was reduced in both female and male lung cancer patients (Figure 2A). According to cancer stage, significant downregulation of TMPRSS2 expression was observed in stage 1, 2, 3 and 4 LUAD and LUSC patients (Figure 2B). In terms of nodal metastasis status, TMPRSS2 expression was also greatly decreased in N0, N1, N2 and N3 in both LUSC and LUAD (Figure 2C). TMPRSS2 expression was lower in tumors from patients in different age groups (21–40, 41–60, 61–80 and 81–100 years) than in normal lung tissues (Supplementary Figure 2A). Moreover, TMPRSS2 expression in Asian, African-American and Caucasian was significantly decreased in LUSC patients (Supplementary Figure 2B). TMPRSS2 expression was dramatically downregulated in Caucasian and African-American LUAD patients (Supplementary Figure 2B). TMPRSS2 expression was similarly reduced in both TP53-mutant and TP53-nonmutant LUAD and LUSC patients (Supplementary Figure 2C). In summary, these results demonstrated that TMPRSS2 expression is significantly correlated with clinicopathological parameters in lung cancer patients.

### TMPRSS2 is an independent predictor of prognosis in lung cancer

**Figure 3:**
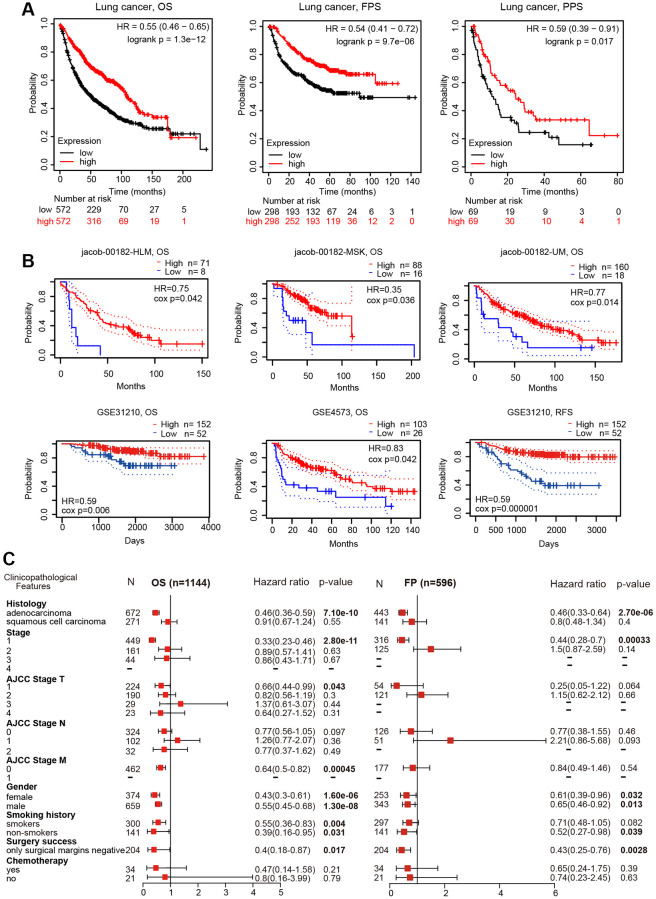
**Prognostic value of TMPRSS2 expression in lung cancer.** (**A**) The OS, FPS and PPS of lung cancer patients were obtained from the Kaplan–Meier plotter database. (**B**) The OS and RFS of lung cancer cohorts obtained through the Prognoscan database. (**C**) Forest plots showing the associations between TMPRSS2 expression and various clinicopathological features of patients with lung cancer.

The impact of TMPRSS2 on the survival of lung cancer patients was analyzed with the PrognoScan and Kaplan–Meier plotter databases. Lung cancer patients with lower TMPRSS2 expression exhibited poor overall survival (OS), postprogression survival (PPS) and first-progression survival (FPS) according to the Kaplan–Meier plotter database (Figure 3A). In addition, the analysis results from the PrognoScan database indicated that decreased TMPRSS2 expression was linked with inferior OS and relapse-free survival (RFS) in different lung cancer cohort samples (Figure 3B). Thus, a low transcriptional level of TMPRSS2 was associated with an unfavorable prognosis.

### Prognostic potential of TMPRSS2 according to different clinicopathological characteristics

To further explore the prognostic potential of TMPRSS2 expression in lung cancer, the relationships between TMPRSS2 expression and the clinical features of lung cancer patients were examined. Intriguingly, reduced TMPRSS2 expression was strongly related with worse OS and FPS in LUAD, but not in LUSC (Figure 3C), and decreased TMPRSS2 expression was obviously linked with OS and FPS in stage 1 lung cancer (Figure 3C). Additionally, there were significant associations between TMPRSS2 expression and poor OS in AJCC stage T-1 and stage M-0 lung cancer patients (Figure 3C). In the analysis by smoking history, downregulation of TMPRSS2 expression contributed to poor OS in both smokers and nonsmokers and poor FPS in nonsmokers (Figure 3C). With respect to sex, low TMPRSS2 expression was strongly linked with worse OS and FPS in female and male lung cancer patients (Figure 3C).

### Conduction of univariate and multivariate Cox hazard regression analysis

**Figure 4:**
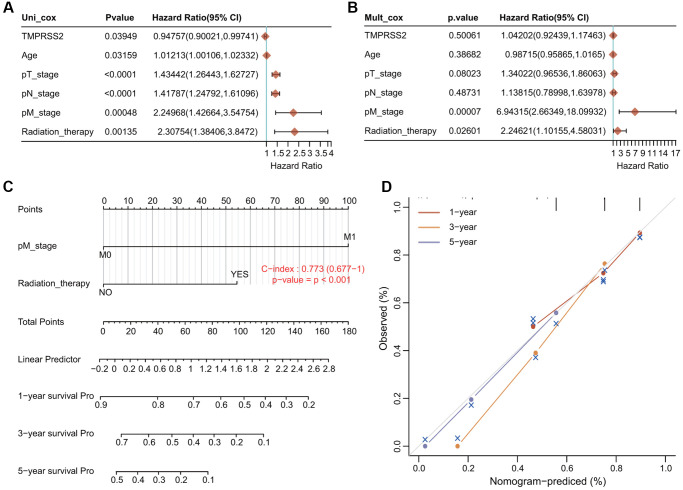
**Establishment and validation of the prognostic nomogram.** (**A**, **B**) Univariate and multivariate Cox regression analysis of clinicopathologic variables and TMPRSS2 in lung cancer. (**C**) Nomogram for predicting the 1-, 3-, and 5-year OS of lung cancer patients. (**D**) Calibration curves of 1-, 3-, and 5-year OS of lung cancer patients.

We conducted univariate Cox and multivariate Cox regression analyses to explore whether TMPRSS2 expression was an independent prognostic factor that correlated with the OS of lung cancer patients. The results of univariate Cox regression analysis indicated that TMPRSS2 expression, age, T stage, N stage, M stage and radiation therapy were obviously correlated with the OS of lung cancer patients (Figure 4A). Moreover, the results of multivariate Cox regression analysis indicated that M stage and radiation therapy showed obvious correlations with the OS of lung cancer patients (Figure 4B). According to these results, TMPRSS2 can serve as an independent prognostic biomarker of OS when adjusted by other related variables.

### Construction of a nomogram model

We then developed a novel nomogram model to predict the 1-, 3-, and 5-year OS rates of lung cancer patients (Figure 4C). The C index (concordance index) of the prognostic nomogram is 0.773 (Figure 4C). The calibration plots for predicting the 1-, 3-, and 5-year OS rates of lung cancer patients also showed good agreement between the predicted and actual survival outcomes (Figure 4D).

### The DNA methylation level and genetic alterations in TMPRSS2 in lung cancer

**Figure 5:**
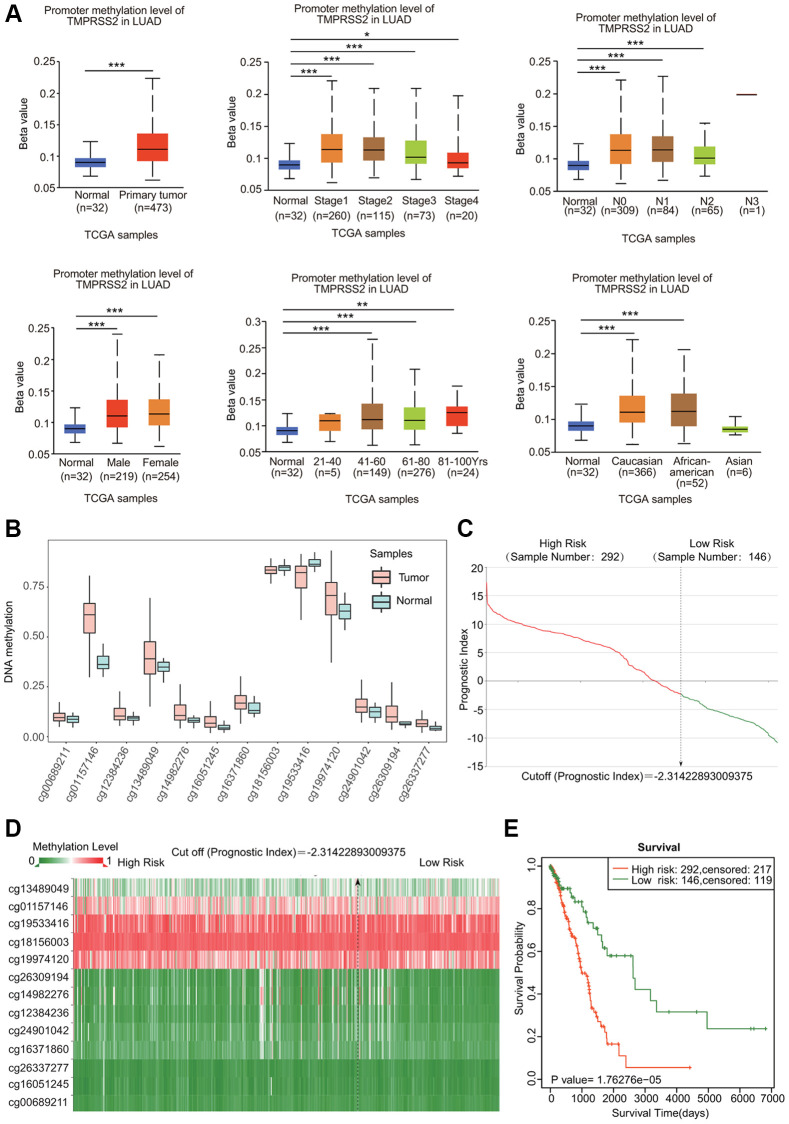
**DNA methylation of TMPRSS2 in LUAD.** (**A**) Associations of DNA methylation of TMPRSS2 with clinicopathological parameters of LUAD. (**B**) Methylation levels of TMPRSS2 in LUAD according to the SurvivalMeth database. (**C**) The distribution of prognostic index in LUAD. (**D**) Heatmap of DNA methylation of TMPRSS2 in LUAD. (**E**) The prognostic potential of DNA methylation of TMPRSS2 in LUAD based on the SurvivalMeth database. ^*^p < 0.05, ^**^p < 0.01, ^***^p < 0.001.

DNA methylation is known to be associated with gene expression and cancer development. We assessed the DNA methylation of TMPRSS2. Both LUAD and LUSC samples showed elevated levels of DNA methylation of TMPRSS2 (Figure 5A; Supplementary Figure 3A). Moreover, the DNA methylation level of TMPRSS2 was also greatly upregulated in LUAD and LUSC patients with different sexes, tumor stages, nodal metastasis statuses, ages and races (Figure 5A; Supplementary Figure 3A). According to the SurvivalMeth database, we also observed increased methylation levels in different CpG sites in the DNA of the TMPRSS2 gene (Figure 5B, 5C; Supplementary Figure 3B, 3C). The heatmap of the DNA methylation results for TMPRSS2 in LUAD and LUSC is shown in Figure 5D and Supplementary Figure 3D. Higher methylation level in CpG sites of the TMPRSS2 gene was linked with worse prognosis in LUAD and LUSC (Figure 5E; Supplementary Figure 3E).

cBioPortal was used to analyze the genetic alterations in TMPRSS2 in lung cancer. Genetic alterations in TMPRSS2 occurred in 1.2% of lung cancer patients (Supplementary Figure 4A). In LUAD, TMPRSS2 was mainly altered by mutation and amplification, whereas TMPRSS2 was mainly altered by deep deletion in LUSC and NSCLC (Supplementary Figure 4B). However, the Kaplan–Meier plotter results indicated that although the survival rate of patients without TMPRSS2 alterations appeared to be worse, there were no significant differences in OS, DFS, PFS or disease-specific survival (DSS) between patients with lung cancer with alterations in TMPRSS2 and those without alterations in TMPRSS2 (Supplementary Figure 4C).

### Key candidate genes and proteins identified from the TMPRSS2 interactive network

A gene–gene interaction network for TMPRSS2 was constructed using the GeneMANIA database. The top three genes significantly correlated with TMPRSS2 were KDM3A, POU2F1 and SLC37A1 (Supplementary Figure 5A). To further estimate the functions of TMPRSS2, a protein–protein interaction (PPI) network was carried out through the STRING database. A total of 10 TMPRSS2-interacting proteins were identified (Supplementary Figure 5B). Among the 11 nodes, the three nodes with the highest degree centrality are AR, ACE2, and TMPRSS4 (Supplementary Figure 5B). Interestingly, two common hub genes were shown from the STRING and GeneMANIA databases: AR and SLC45A3. We then assessed the correlations between TMPRSS2 and these two proteins in the TIMER and GEPIA2 databases. TMPRSS2 expression was correlated with AR and SLC45A3 in LUAD and only correlated with AR in LUSC (Supplementary Figure 5C, 5D). We then examined the relationship between TMPRSS2 and other targets for COVID-19 therapy, including ACE2, AXL, CTSL and FURIN. TMPRSS2 was positively correlated with ACE2 and AXL in LUAD and LUSC but negatively associated with CTSL in LUAD (Supplementary Figure 5E).

### KEGG and GO analyses of TMPRSS2

**Figure 6:**
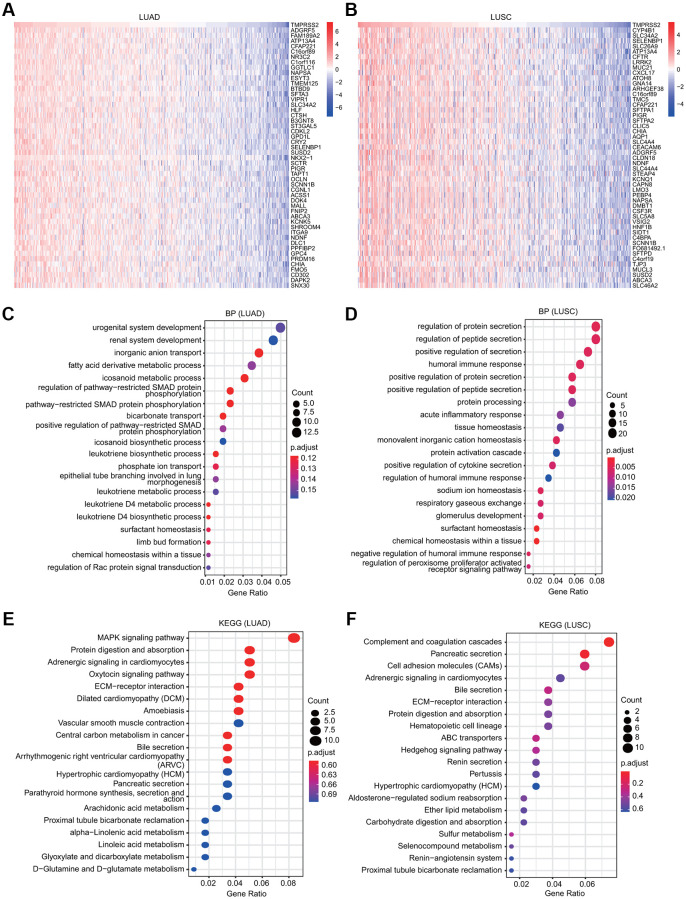
**GO and KEGG analyses for TMPRSS2 in lung cancer.** (**A**, **B**) Heatmaps showing the top 50 genes that were positively correlated with TMPRSS2 in LUAD and LUSC. (**C**, **D**) Top 20 enrichment terms in the BP category in LUAD and LUSC. (**E**, **F**) Top 20 pathways enriched in the KEGG analysis in LUAD and LUSC.

The functions of TMPRSS2 and the genes significantly associated with TMPRSS2 alterations were predicted by GO and KEGG analyses. A total of 300 coexpressed TMPRSS2 genes were used, and the top fifty genes that were positively and negatively associated with TMPRSS2 in LUSC and LUAD are shown (Figure 6A, 6B and Supplementary Figure 6A, 6B). Furthermore, the top 20 significant terms of GO enrichment analysis are presented. Regarding the biological process (BP) terms, the results showed that urogenital and renal system development and various metabolic processes were associated with TMPRSS2 in LUAD; multiple immune-related pathways, including humoral immune response, acute inflammatory response, positive regulation of cytokine secretion, and regulation of humoral immune response, were significantly correlated with TMPRSS2 in LUSC (Figure 6C, 6D). Regarding the molecular function (MF) terms, the results suggested that enzyme inhibitor activity, coenzyme binding and inorganic anion transmembrane transporter activity were associated with TMPRSS2 in LUAD; anion transmembrane transporter activity, carbohydrate binding and gated channel activity were associated with TMPRSS2 in LUSC (Supplementary Figure 6C, 6D). Regarding the cellular component (CC) terms, the results suggested that the apical part of the cell, collagen-containing extracellular matrix, and apical plasma membrane were related with TMPRSS2 in both LUAD and LUSC (Supplementary Figure 6E, 6F). Additionally, KEGG analysis results suggested that TMPRSS2 was involved in adrenergic signaling in cardiomyocytes, ECM-receptor interaction, hypertrophic cardiomyopathy (HCM), and bile secretion in lung cancer (Figure 6E, 6F).

### GSEA revealed TMPRSS2-associated signaling pathways

**Figure 7:**
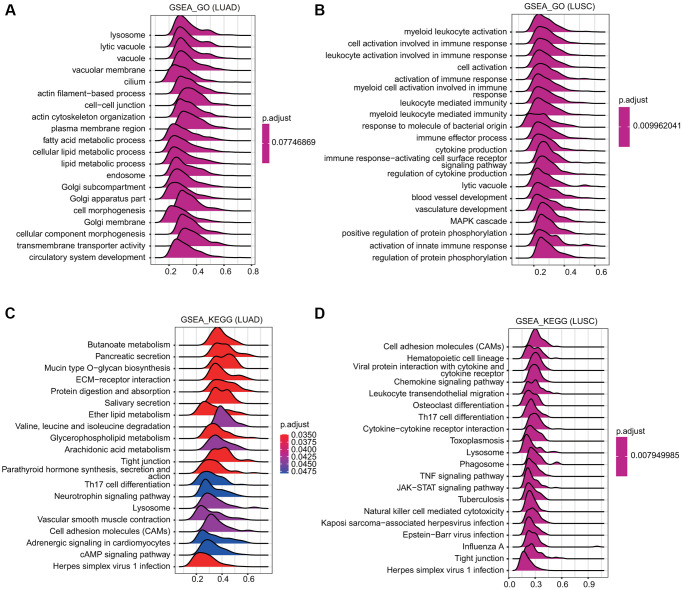
**Merged enrichment plots obtained by GSEA.** (**A**, **B**) Merged plots indicating the signaling pathways associated with TMPRSS2 expression according to GO analyses in LUAD and LUSC. (**C**, **D**) Merged plots indicating the signaling pathways associated with TMPRSS2 expression according to KEGG analyses in LUAD and LUSC.

GSEA was conducted to examine the TMPRSS2-associated signaling pathways that were differentially activated in lung cancer. The outcome implied that regarding the GO terms in LUSC, the top twenty signaling pathways affected by TMPRSS2 were mainly enriched in immune response-associated activities, including myeloid leukocyte activation, leukocyte activation involved in the immune response, cell activation involved in the immune response, activation of the immune response, leukocyte mediated-immunity, immune effector process, cytokine production, immune response-activating cell surface receptor signaling pathway, and activation of the innate immune response (Figure 7A, 7B). Similarly, regarding the KEGG terms, the GSEA results indicated various immune functional gene sets that were enriched in both LUAD and LUSC, including Th17 cell differentiation, cytokine–cytokine receptor interaction, herpes simplex virus 1 infection, and the TNF signaling pathway (Figure 7C, 7D). These findings demonstrate that TMPRSS2 plays a critical role in the TME (tumor microenvironment).

We further identified the associations between TMPRSS2 and human diseases to assist drug discovery using the Open Targets platform. We found that TMPRSS2 was associated with various human diseases, such as cardiovascular disease, endocrine system diseases, immune system disease, respiratory or thoracic disease, infectious diseases (COVID-19 and severe acute respiratory syndrome) (Supplementary Figure 7A) and cancer or benign tumors (prostate carcinoma, gastric adenocarcinoma, colon adenocarcinoma, rectal adenocarcinoma, small cell lung carcinoma, etc) (Supplementary Figure 7B).

### The correlations between TMPRSS2 expression and immune cell infiltration in lung cancer

**Figure 8:**
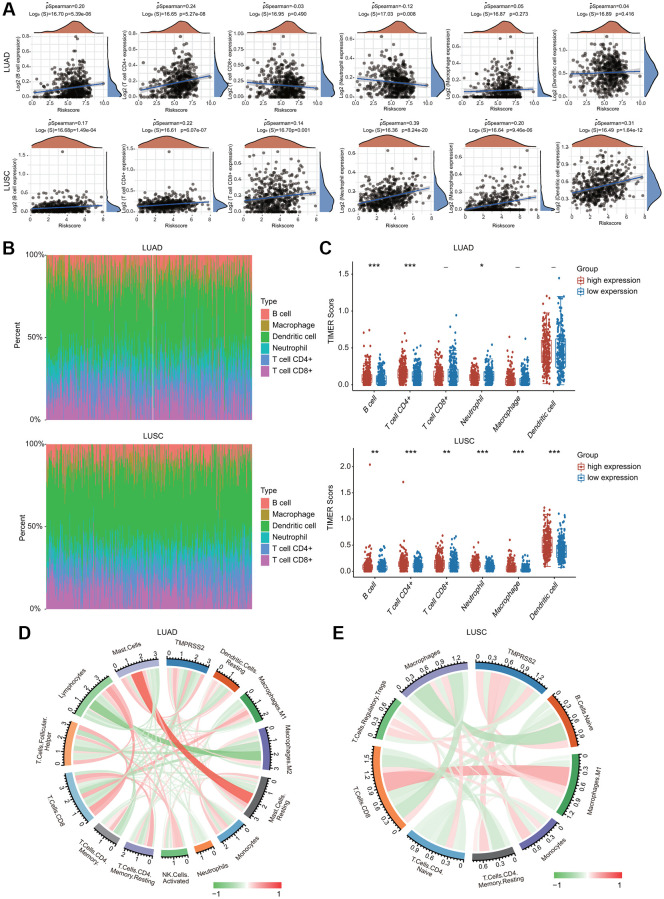
**Association between TMPRSS2 expression and infiltration levels of immune cells in lung cancer.** (**A**) TMPRSS2 expression was significantly correlated with the infiltration levels of various immune cells in LUAD and LUSC in the TIMER database. (**B**, **C**) TMPRSS2 expression was significantly associated with the infiltration of immune cells in LUAD and LUSC according to the CIBERSORT algorithm. (**D**) Heatmap of the correlation of TMPRSS2 and immune checkpoints in LUAD. (**E**) Heatmap of the correlation of TMPRSS2 and immune checkpoints in LUSC. ^*^p < 0.05, ^**^p < 0.01, ^***^p < 0.001.

The potential immunological correlations of TMPRSS2 and immune cell infiltration were investigated. TMPRSS2 expression was significantly correlated with the infiltration levels of B cells, CD4+ T cells and neutrophils in LUAD (Figure 8A). TMPRSS2 expression was positively and significantly linked with the infiltrating levels of all six types of immune cells in LUSC (Figure 8A). According to TMPRSS2 expression, lung cancer patients were divided into low- and high-expression groups. The percentage abundance of tumor infiltrating immune cells in each sample is indicated using multiple colors for various types of immune cells using TIMER (Figure 8B). We observed that the infiltrating levels of B cells and CD4+ T cells were enhanced in the TMPRSS2 high-expression group compared with the low-expression group in LUAD (Figure 8C). Moreover, the infiltrating levels of CD4+ T cells, CD8+ T cells, B cells, neutrophils, macrophages, and dendritic cells were increased in the TMPRSS2 high-expression group compared with the low-expression group in LUSC (Figure 8C). The correlations between TMPRSS2 expression and immune cell infiltration were also confirmed by the established computational resource CIBERSORT. Notably, TMPRSS2 was positively associated with the infiltration abundances of resting dendritic cells, dendritic cells, M2 macrophages, resting mast cells, mast cells, monocytes, and resting CD4 memory T cells but negatively associated with the infiltration abundances of lymphocytes, M0 macrophages, M1 macrophages, neutrophils, activated mast cells, activated NK cells, resting NK cells, plasma cells, activated memory CD4 T cells, CD8 T cells, follicular helper T cells and gamma delta T cells in LUAD (Figure 8D and Supplementary Figure 8A, 8B). Additionally, TMPRSS2 was positively associated with the infiltration abundances of naïve B cells, lymphocytes, resting mast cells, monocytes, Treg cells, neutrophils, and resting memory CD4 T cells but negatively correlated with the infiltration abundances of macrophages, M0 macrophages, M1 macrophages, CD8 T cells, naïve CD4 T cells, activated memory CD4 T cells, and eosinophils in LUSC (Figure 8E and Supplementary Figure 9A, 9B).

### Correlations between TMPRSS2 and immune cell marker sets

To further explore the association between TMPRSS2 and these multiple populations of infiltrating immune cells, we established correlation between TMPRSS2 and different marker sets of multiple immune cells through the TIMER and GEPIA2 databases. TMPRSS2 mRNA expression was significantly correlated with most diverse immune cell markers in lung cancer (Tables 1 and 2).

**Table 1:** Correlations between TMPRSS2 and various gene markers of immune cells in TIMER.

**Description**	**Gene markers**	**LUAD**				**LUSC**			
		**None**		**Purity**		**None**		**Purity**	
		***P***	**Cor**	***P***	**Cor**	***P***	**Cor**	***P***	**Cor**
**T cell**	CD2	0.907	0.005	0.418	0.037	^***^	0.233	^**^	0.133
	CD3D	0.089	−0.075	0.198	−0.058	^***^	0.208	^*^	0.1
**B cell (general)**	CD3E	0.84	0.009	0.313	0.046	^***^	0.259	^***^	0.161
	CD19	0.285	0.047	0.0506	0.088	^***^	0.289	^***^	0.196
	CD79A	0.685	0.018	0.214	0.056	^***^	0.269	^***^	0.164
**CD8+ T cell**	CD8A	^***^	−0.154	^**^	−0.132	^***^	0.147	0.164	0.064
	CD8B	^***^	−0.156	^**^	−0.134	^**^	0.14	^*^	0.092
**TAM**	IL10	0.736	−0.015	0.889	0.006	^***^	0.277	^***^	0.193
	CD68	0.557	−0.026	0.992	0	^***^	0.251	^**^	0.143
**Monocyte**	CCL2	0.614	0.022	0.520	0.029	^***^	0.224	^**^	0.148
	CSF1R	^**^	0.12	^***^	0.152	^***^	0.312	^***^	0.206
	CD86	0.966	−0.002	0.512	0.03	^***^	0.3	^***^	0.196
**M2**	CD163	0.415	−0.036	0.77	−0.013	^***^	0.298	^***^	0.2
	MS4A4A	0.924	0.004	0.486	0.031	^***^	0.267	^***^	0.163
**M1**	VSIG4	0.825	−0.01	0.758	0.014	^***^	0.249	^***^	0.151
	PTGS2	0.265	0.049	0.352	0.042	^***^	0.266	^***^	0.223
	IRF5	^*^	0.097	^**^	0.12	0.897	−0.006	−0.024	0.598
**Natural killer cell**	KIR2DS4	^*^	−0.113	^*^	−0.116	^**^	0.129	^*^	0.104
	KIR3DL3	^***^	−0.234	^***^	−0.243	0.473	−0.032	0.280	−0.05
	KIR3DL2	^***^	−0.169	^***^	−0.173	^**^	0.126	0.0749	0.082
**Neutrophils**	KIR3DL1	^*^	−0.103	^*^	−0.104	^**^	0.141	^*^	0.093
	KIR2DL4	^***^	−0.391	^***^	−0.386	0.529	0.028	0.465	−0.034
	KIR2DL3	^***^	−0.229	^***^	−0.214	^*^	0.107	0.107	0.074
	KIR2DL1	^**^	−0.131	^**^	−0.13	^**^	0.124	^*^	0.09
	CCR7	^***^	0.233	^***^	0.295	^***^	0.339	^***^	0.26
	CEACAM8	^***^	0.391	^***^	0.394	^***^	0.175	^***^	0.172
	ITGAM	^***^	0.156	^***^	0.185	^***^	0.358	^***^	0.266
**Dendritic cell**	HLA-DPB1	^***^	0.307	^***^	0.358	^***^	0.358	^***^	0.266
	HLA-DRA	^***^	0.231	^***^	0.277	^***^	0.299	^***^	0.203
	HLA-DQB1	^***^	0.284	^***^	0.327	^***^	0.199	^*^	0.094
	HLA-DPA1	^***^	0.283	^***^	0.333	^***^	0.327	^***^	0.237
	CD1C	^***^	0.49	^***^	0.516	^***^	0.399	^***^	0.316
	NRP1	^***^	0.167	^***^	0.167	^***^	0.209	^**^	0.12
	ITGAX	0.0712	0.08	^**^	0.12	^***^	0.354	^***^	0.248

**Table 2:** Correlations between TMPRSS2 and various gene markers of immune cells in GEPIA2.

**Description**	**Gene markers**	**LUAD**		**LUSC**	
		***P***	**Cor**	***P***	**Cor**
**TAM**	CCL2	0.15	0.065	^***^	0.23
	CD68	0.51	0.03	^***^	0.24
	IL10	0.37	0.041	^***^	0.27
**T cell (general)**	CD2	0.81	0.011	^***^	0.23
	CD3D	0.077	−0.08	^***^	0.2
	CD3E	0.66	0.02	^***^	0.25
**B cell**	CD79A	0.68	−0.019	^***^	0.25
	CD19	0.59	0.025	^***^	0.26
**CD8+ T cell**	CD8A	^**^	−0.15	^**^	0.14
	CD8B	^***^	−0.15	^**^	0.13
**Monocyte**	CD86	0.44	0.035	^***^	0.29
	CSF1R	^***^	0.16	^***^	0.31
**M1**	PTGS2	0.092	0.077	^***^	0.27
	IRF5	^**^	0.13	0.78	−0.013
**M2**	VSIG4	0.52	0.029	^***^	0.24
	MS4A4A	0.28	0.049	^***^	0.26
	CD163	^*^	−0.097	^***^	0.27
**Neutrophils**	ITGAM	^***^	0.22	^***^	0.36
	CCR7	^***^	0.25	^***^	0.34
	CEACAM8	^***^	0.46	^***^	0.22
**Natural killer cell**	KIR2DS4	^**^	−0.14	^**^	0.12
	KIR3DL3	^***^	−0.25	0.93	0.0041
	KIR3DL2	^***^	−0.15	^**^	0.14
	KIR3DL1	^*^	−0.11	^***^	0.18
	KIR2DL4	^***^	−0.39	0.57	0.026
	KIR2DL3	^***^	−0.24	^*^	0.1
	KIR2DL1	^***^	−0.16	^**^	0.13
**Dendritic cell**	HLA-DQB1	^***^	0.23	^**^	0.12
	HLA-DPB1	^***^	0.33	^***^	0.36
	HLA-DRA	^***^	0.27	^***^	0.3
	HLA-DPA1	^***^	0.32	^***^	0.32
	ITGAX	^**^	0.12	^***^	0.34
	NRP1	^***^	0.22	^***^	0.24
	CD1C	^***^	0.5	^***^	0.4

**Figure 9:**
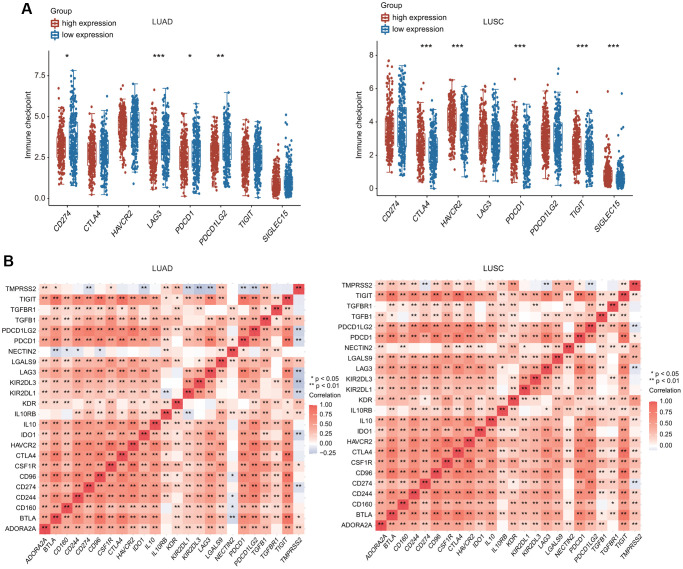
**Relationship between TMPRSS2 expression and immune checkpoint genes.** (**A**) The expression of multiple immune checkpoint genes between TMPRSS2 high-expression group and TMPRSS2 low-expression group in LUAD and LUSC. (**B**) Heatpmap of correlations between TMPRSS2 expression and immune checkpoint genes in LUAD and LUSC.

Furthermore, we investigated the relationship between TMPRSS2 and various types of T cells. TMPRSS2 expression was strongly related with 37 of 54 T cell markers in LUAD and with 35 of 54 T cell markers in LUSC (Table 3). Significantly lower expression of CD274 (PD-1), PDCD-1 (PD-L1), PDCD1LG2, and LAG3 was observed in the TMPRSS2 high-expression group compared with the TMPRSS2 low-expression group in LUAD (Figure 9A). In contrast, higher expression of CTLA4, HAVCR2, PDCD1, TIGIT and SIGLEC15 was found in the TMPRSS2 high-expression group compared with the TMPRSS2 low-expression group in LUSC (Figure 9A). The correlations between TMPRSS2 and various immune checkpoints, including CTLA-4, PD-1 and PD-L1, were then assessed. TMPRSS2 expression was positively and strongly associated with ADORA2A, IL10RB, LAGLS9, TGFB1 and KDR but negatively associated with IDO1, LAG3, PD-1, PDCD1, PDCD1LG2, KIR2DL1 and KIR2DL3 in LUAD (Figure 9B). In contrast, TMPRSS2 expression was positively associated with most immune checkpoints in LUSC (Figure 9B). We also found that TMPRSS2 expression was significantly negatively correlated with TMB in LUAD and LUSC. Additionally, TMPRSS2 expression was weakly correlated with MSI in LUSC (Supplementary Figure 10A, 10B).

**Table 3:** Correlations between TMPRSS2 and gene markers of diverse types of T cell in TIMER.

**Description**	**Gene markers**	**LUAD**				**LUSC**			
		**None**		**Purity**		**None**		**Purity**	
		***P***	**Cor**	***P***	**Cor**	***P***	**Cor**	***P***	**Cor**
**Th1**	TNF	^***^	0.173	^**^	0.124	0.9	0.006	0.128	−0.07
	IFNG	0.105	0.072	^*^	0.089	^***^	0.242	^**^	0.149
	TBX21	0.401	−0.037	0.807	−0.011	^***^	0.242	^***^	0.152
	STAT1	^***^	−0.222	^***^	−0.211	0.620	0.022	0.329	−0.045
	STAT4	^*^	0.101	^**^	0.13	^***^	0.341	^***^	0.249
**Th1-like**	HAVCR2	0.492	−0.03	0.907	−0.005	^***^	0.256	^***^	0.153
	IFNG	^***^	−0.323	^***^	−0.316	0.965	0.002	0.164	−0.064
	CXCR3	0.534	0.027	0.251	0.052	^***^	0.272	^***^	0.185
	CXCL13	^*^	−0.089	0.101	−0.074	^***^	0.171	0.131	0.69
	BHLHE40	^***^	0.156	^**^	0.142	^***^	0.301	^***^	0.257
	CD4	^***^	0.163	^***^	0.212	^***^	0.35	^***^	0.252
**Th2**	BCL6	^***^	0.303	^***^	0.304	^***^	0.175	^***^	0.225
	STAT5A	^***^	0.163	^***^	0.2	^***^	0.359	^***^	0.274
	GATA3	0.620	−0.022	0.784	−0.012	^***^	0.149	0.073	0.082
	STAT3	^***^	0.343	^***^	0.353	^***^	0.336	^***^	0.308
	STAT6	^***^	0.372	^***^	0.382	^***^	0.259	^***^	0.264
	IL13	0.0538	0.085	^*^	0.095	^***^	0.187	^**^	0.128
	IL21	^***^	−0.149	^***^	−0.137	^**^	0.142	0.1	0.075
	IL17A	0.0539	−0.085	0.182	−0.06	0.612	−0.023	0.0628	−0.085
**Treg**	STAT5B	^***^	0.237	^***^	0.247	0.256	0.051	0.201	0.059
	FOXP3	0.7	0.017	0.310	0.046	^***^	0.253	^***^	0.151
	TGFB1	^***^	0.266	^***^	0.291	^**^	0.143	0.131	0.069
	CCR8	0.128	0.067	^*^	0.096	^***^	0.254	^**^	0.164
**Resting Treg**	FOXP3	0.7	0.017	0.310	0.046	^***^	0.253	^***^	0.151
	IL2RA	^**^	−0.141	^**^	−0.135	^***^	0.206	^*^	0.102
**Effector Treg T cell**	FOXP3	0.7	0.017	0.310	0.046	^***^	0.253	^***^	0.151
	CTLA4	0.298	−0.046	0.594	−0.024	^***^	0.186	0.0992	0.076
	CCR8	0.128	0.067	^*^	0.096	^***^	0.254	^**^	0.164
	TNFRSF9	^*^	−0.109	^*^	−0.1	^***^	0.165	0.193	0.06
**Effector T cell**	FGFBP2	^***^	0.194	^***^	0.206	0.0532	−0.086	0.385	−0.04
	FCGR3A	^**^	−0.141	^**^	−0.122	^***^	0.187	0.0722	0.082
	CX3CR1	^***^	0.454	^***^	0.474	^***^	0.342	^***^	0.272
**Naïve T cell**	CCR7	^***^	0.233	^***^	0.295	^***^	0.339	^***^	0.26
	SELL	^***^	0.152	^***^	0.192	^***^	0.356	^***^	0.262
	TCF7	0.0732	0.079	^*^	0.097	^***^	0.197	^**^	0.136
	LEF1	0.236	0.052	0.195	0.058	^*^	−0.1	0.142	−0.067
	PDCD1	^**^	−0.126	^*^	−0.111	^***^	0.214	^**^	0.121
	DUSP4	^***^	−0.301	^***^	−0.296	^***^	0.174	^*^	0.113
	GZMK	0.843	0.009	0.350	0.042	^***^	0.23	^**^	0.134
	GZMA	^***^	−0.24	^***^	−0.234	^*^	0.106	0.761	0.014
	IFNG	^***^	−0.323	^***^	−0.316	0.965	0.002	0.164	−0.064
	CD69	^**^	0.133	^***^	0.177	^***^	0.295	^***^	0.198
	ITGAE	^***^	−0.199	^***^	−0.189	^***^	−0.166	^*^	−0.117
	CXCR6	0.0849	−0.076	0.254	−0.052	^***^	0.22	^**^	0.13
	MYADM	^*^	0.1	^**^	0.117	^***^	0.194	^**^	0.123
**General memory T cell**	CCR7	^***^	0.233	^***^	0.295	^***^	0.339	^***^	0.26
	SELL	^***^	0.152	^***^	0.192	^***^	0.356	^***^	0.262
	IL7R	^**^	0.126	^***^	0.156	^***^	0.255	^***^	0.152
**Exhausted T cell**	HAVCR2	0.492	−0.03	0.907	−0.005	^***^	0.256	^***^	0.153
	TIGIT	0.209	−0.055	0.494	−0.031	^***^	0.221	^**^	0.121
	LAG3	^***^	−0.211	^***^	−0.196	0.0521	0.087	0.947	0.003
	PDCD1	^**^	−0.126	^*^	0−0.11	^***^	0.214	^**^	0.121
	CXCL13	^*^	−0.089	0.101	−0.074	^***^	0.171	0.131	0.069
	LAYN	0.571	0.025	0.387	0.039	0.915	−0.005	0.661	−0.02

### Prognostic analysis of TMPRSS2 expression according to immune cell infiltration in lung cancer

**Figure 10:**
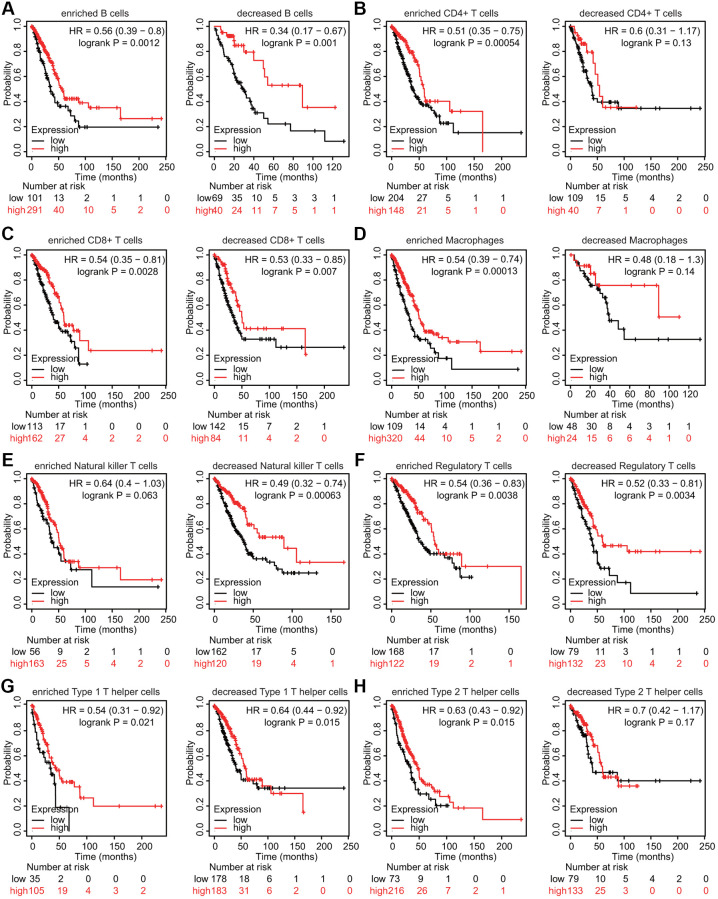
**Kaplan–Meier survival curves based on high and low expression levels of TMPRSS2 in immune cell subgroups in LUAD.** (**A**–**H**) The relationship between TMPRSS2 expression and the OS rate in different immune cell subgroups of LUAD patients was explored.

We also estimated whether TMPRSS2 influenced the prognosis of patients with lung cancer through its effects on immune cell infiltration. Prognostic analysis according to TMPRSS2 expression in patients stratified by populations of related immune cell subgroups was carried out. The low expression of TMPRSS2 in the LUAD patient cohorts with increased CD4+ T cells, increased macrophages, decreased NK T cells and increased Th2 cells was linked to worse prognosis (Figure 10B, 10D, 10E, 10H). In addition, a significant correlation between low TMPRSS2 expression and inferior prognosis was observed in the cohorts with either increased or decreased CD8+ T cells, B cells, Treg cells, and Th1 cells populations in LUAD (Figure 10A, 10C, 10F, 10G). Moreover, the high expression of TMPRSS2 in the LUSC patient cohorts with decreased B cells, CD4+ memory T cells, CD8+ T cells, Th1 cells and Th2 cells exhibited worse OS (Supplementary Figure 11A, 11B, 11C, 11G, 11H). In contrast, no significant correlations between TMPRSS2 expression and prognosis were observed in the cohorts with either increased or decreased macrophage, NK T cell, or Treg cell populations in LUSC patients (Supplementary Figure 11D, 11E, 11F). These findings suggest that TMPRSS2 expression affects the prognosis of patients with lung cancer partially through immune cell infiltration.

### TMPRSS2 expression was downregulated during SARS-CoV infection

**Figure 11:**
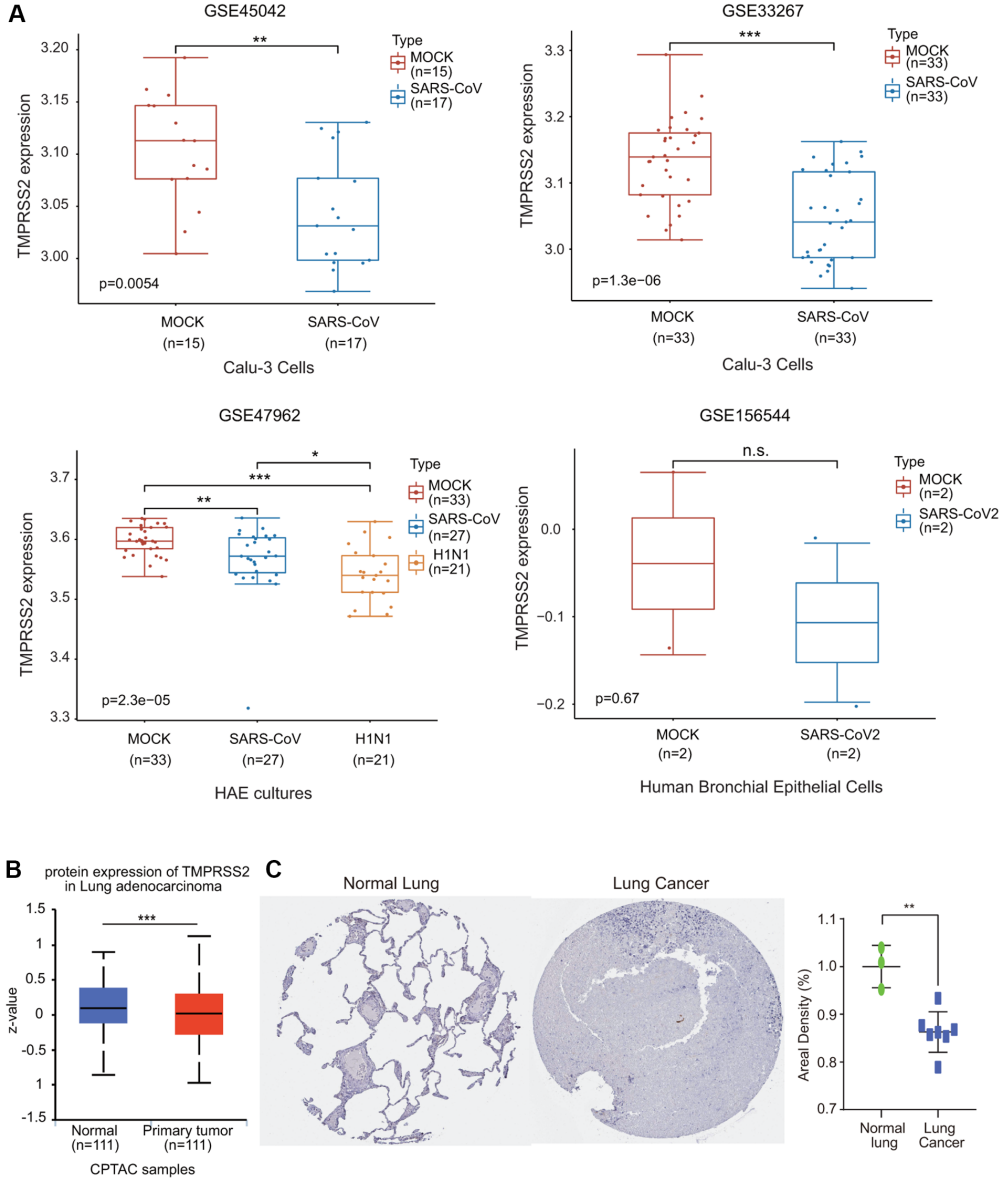
**The alteration of TMPRSS2 expression during SARS-CoV-2 infection.** (**A**) The change in TMPRSS2 expression in the GSE45042 (mock, *n* = 15; SARS-CoV, *n* = 17), GSE33267 (mock, *n* = 33; SARS-CoV, *n* = 33), GSE17962 (mock, *n* = 33; SARS-CoV, *n* = 27; H1N1, *n* = 21), and GSE156544 (mock, *n* = 2; SARS-CoV2, *n* = 2) datasets. (**B**) TMPRSS2 protein levels in lung cancer tissues (*n* = 111) and normal tissues (*n* = 111) from the UALCAN database. (**C**) TMPRSS2 protein level in lung cancer and normal tissues from the HPA database. The staining was quantified (normal lung tissue, *n* = 3; lung cancer, *n* = 7). ^*^p < 0.05, ^**^p < 0.01, ^***^p < 0.001.

We then investigated the effect of coronavirus on TMPRSS2 expression. We collected four GEO databases, GSE33267, GSE47962, GSE45042 and GSE156544, to assess TMPRSS2 expression during SARS-CoV infection. TMPRSS2 expression was significantly reduced in Calu-3 cells and HAE cultures infected with SARS-CoV (Figure 11A). Additionally, TMPRSS2 expression was decreased in human bronchial epithelial cells infected with SARS-CoV2, although the difference was not significant difference (Figure 11A).

We also examined the protein expression level of TMPRSS2 in lung cancer using the UALCAN database. The protein level of TMPRSS2 was also much lower in lung cancer tissues than in normal lung tissues (Figure 11B). We also retrieved the IHC staining data from the HPA database. Normal lung tissues exhibited moderate TMPRSS2 staining, while TMPRSS2 could not be detected in most lung cancer tissues (Figure 11C). The results indicated that TMPRSS2 had significantly lower protein expression in lung cancer. These findings further confirmed the decreased expression of TMPRSS2 in lung cancer.

## DISCUSSION

COVID-19 has proven to be a dangerous and far-reaching disease, and the number of infections and deaths worldwide continues to drastically rise worldwide [1–3]. Although most patients eventually recover, the world has not recently experienced another large-scale destruction in such a short period of time. The long-term effects of this virus are currently unclear, although it is thought that many patients will have serious sequelae from this infection. Thus, in addition to preventing infection, research on the factors that determine susceptibility to COVID-19 infection and the mechanisms behind these factors are critical for the control of SARS-CoV-2.

Lung cancer patients are at high risk of COVID-19 infection because the lung is the major target organ of SARS-CoV-2 infection [7]. COVID-19 appears to have a worse prognosis in cancer patients who have been admitted to the intensive care unit and in those requiring mechanical ventilation and increased mortality, especially in those who have recently received surgery or chemotherapy. SARS-CoV-2 infects humans by binding to ACE2, which is a transmembrane endopeptidase that can cleave angiotensin 1 and 2 and is expressed by epithelial cells of multiple organs, including the airway [1–5]. The cofactor that promotes SARS-CoV-2 infection is TMPRSS2, which could cleave the SARS-CoV-2 spike protein and possibly the protease furin. Additionally, TMPRSS2 expression is associated with the infectivity of various respiratory viruses. TMPRSS2-KO mice showed stronger resistance to influenza [17–19]. During the H1N1 epidemic in 2009, the TMPRSS2 variant that resulted in increased expression was associated with increased human susceptibility to influenza infection [20]. Previous study suggested that camostat, a serine and cysteine protease inhibitor of TMPRSS2, can partially but significantly block SARS-CoV infection, and the combination with alostatin (a cathepsin inhibitor) could significantly enhance the antiviral effect of camostat [21]. Understanding TMPRSS2 expression in lung cancer patients and its relationship with prognosis may help clarify why cancer patients are more likely to be infected with SARS-CoV-2 and help determine whether lung cancer immunotherapy may change susceptibility to SARS-CoV-2 infection.

When considering the harmful consequences of cancer and COVID-19 disease, an initial hypothesis is that the cancer tissue itself may have higher expression levels of related genes, allowing the virus to enter. However, we found that TMPRSS2 expression does not support this hypothesis in the present study. In contrast to our speculation, TMPRSS2 expression in lung cancer tissues was generally downregulated (Figures 1 and 11). Nevertheless, in the present study, the GSEA results revealed that TMPRSS2 was associated with influenza A, herpes simplex virus 1 infection and Epstein–Barr virus infection, indicating that TMPRSS2 indeed plays a role in viral infection (Figure 7). Previous studies have shown that TMPRSS2 expression was significantly lower in nasopharyngeal swabs of SARS-CoV-2-infected patients than in those of healthy people and patients with other viral acute respiratory diseases [22]. Furthermore, the low TMPRSS2 expression predicted a short survival time in patients with lung cancer (Figure 3). We also evaluated the prognostic value of TMPRSS2 for lung cancer patients by performing Cox regression analyses and prognostic nomograms based on the correlation between TMPRSS2 expression and OS in lung cancer (Figure 4). These observations support that TMPRSS2 is related to carcinogenesis and may function as a promising candidate biomarker for predicting the prognosis of lung cancer. In fact, a pan-cancer analysis identified that both TMPRSS2 and ACE2 were commonly expressed at low levels in cancers compared with matched individuals [14]. A recent study using single-cell RNA-seq data demonstrated that TMPRSS2 is highly expressed in colorectal epithelial tissues and colorectal cancer [23]. More importantly, colorectal cancer patients with SARS-CoV-2 infection exhibited higher rates of lymphopenia, higher levels of hypersensitive C-reactive protein and a higher death rate than COVID-19 patients without colorectal cancer [23]. In contrast, TMPRSS2 expression was downregulated in head and neck cancer and oral squamous cell carcinoma [24–26]. Decreased TMPRSS2 expression was correlated with TP53 mutation and worse OS and DFS in head and neck cancer patients. Knockdown of mutant p53 greatly increased TMPRSS2 expression in head and neck cancer cells, indicating that p53 may modulate TMPRSS2 expression [24]. Moreover, a group of microRNAs was negatively associated with TMPRSS2 expression, indicating that TMPRSS2 expression may be regulated at the posttranscriptional level [24]. In addition, TMPRSS2 expression was also downregulated in tumor tissues in head and neck cancer patients with COVID-19 compared with matched normal individuals [24]. Accumulating evidence indicates that TMPRSS2 plays an important role in the oncogenesis of prostate cancer [27, 28]. Normally, TMPRSS2 is mainly expressed on the luminal side of the prostatic epithelium but is significantly upregulated in malignant prostatic cells and tissues [29]. Increased TMPRSS2 expression correlated with the poor survival of prostate cancer patients. TMPRSS2 promotes prostate oncogenesis not only through elevated expression but also though aberrant cellular localization that induces the loss of epithelial polarity [30]. High levels of TMPRSS2 also facilitate the tumor growth, progression, invasion and metastasis by modulating the activation of matriptase and the integrity of the ECM network. Moreover, inhibition of apoptosis was found in TMPRSS2-ERG-positive prostate cancer cells. This finding may be due to the destruction of the intracellular death domain and/or the corresponding receptor. A recent study identified HAI-2, a cognate inhibitor of TMPRSS2, as mediating the proteolytic activity of TMPRSS2 to inhibit the invasion and metastasis of prostate cancer [31]. In particular, the most common chromosomal aberration in prostate cancer is the fusion of erythroblast-specific-related gene (ERG) and the 5′-UTR of TMPRSS2 [32, 33]. Overexpression of ERG has been found in approximately 40%–50% of primary prostate cancers. Androgens and androgen receptors regulate TMPRSS2 and ACE2 expression [27, 28]. Men with higher androgen receptor transcriptional activity have a higher risk of TMPRSS2-ERG fusion-positive prostate cancer. In a study of 9280 COVID-19 patients from 68 hospitals in northeastern Italy, the researchers found that patients with prostate cancer and patients not treated with androgen deprivation were more susceptible to SARS-CoV-2 infection than patients treated with androgen deprivation, which would reduce the expression of TMPRSS2 [34]. Thus, prostate cancer patients with anti-AR treatment may be less susceptible to SARS-CoV-2 infection. Anti-AR therapy can be used as a therapeutic strategy and preventive option in patients with prostate cancer to inhibit the entry of viruses [35].

Based on our GO and KEGG analysis results, various metabolic pathways, such as fatty acid derivative metabolic process, icosanoid metabolic process, icosanoid biosynthetic process, leukotriene biosynthetic/metabolic process, leukotriene D4 biosynthetic/metabolic process, arachidonic acid metabolism, linolenic acid metabolism, glyoxylate and dicarboxylate metabolism, D-glutamine and D-glutamate metabolism, sulfur metabolism, and selenocompound metabolism, were significantly associated with TMPRSS2 in lung cancer (Figure 6). Consistent with the above findings, our GSEA results also suggest that TMPRSS2 may affect multiple metabolic processes, including fatty acid metabolic processes, lipid metabolic processes, butanoate metabolism, ether lipid metabolism, glycerophospholipid metabolism, and arachidonic acid metabolism (Figure 7). However, the relationships between TMRPSS2 and metabolism and SARS-CoV-2 infection are unclear and deserve further exploration.

COVID-19 also has a strong immune component, and its poor prognosis has recently been thought to be related to cytokine storms and the hyperinflammatory immune system [36, 37]. However, whether TMPRSS2 is involved in regulating antitumor immunity and its clinical significance in lung cancer remain unknown. Adaptive immunity after SARS-CoV-2 infection is necessary for effective virus clearance [38]. Because B and T cells respond quickly to infection and play a key role in defending against viral infection, systematic studies of changes in B and T cells in patients with COVID-19 will be important to reveal the immune response to SARS-COV-2 infection and will also provide insights for the diagnosis and treatment of COVID-19. In SARS-CoV-infected patients, the acute phase of infection was correlated with a severe reduction in the number of T cells in the blood, with a sharp reduction in the number of CD4 and CD8 T cells compared with that in healthy controls [39, 40]. These findings imply that SARS-CoV infection can impair cellular immunity early in the disease course. By analyzing blood samples from COVID-19 patients and healthy donors, it was demonstrated that TfH (follicular helper CD4 T cells) and GCB (germinal center B) cells were significantly increased in patients with mild or moderate symptoms, while patients with severe COVID-19 showed lymphocyte dysfunction characterized by the severe depletion of CD4+ lymphocytes and subsequent B-cell lymphopenia [41]. In addition, using single-cell RNA sequencing, CD8+ and CD4+ T cells were markedly decreased, while B cells were significantly increased during the recovery period of COVID-19 [41, 42]. Overall, these findings provide a preliminary understanding of the phenotypes of the T cell and B cell subtypes related to COVID-19.

In this study, KEGG and GO analyses and GSEA indicated that various immune-related pathways, such as myeloid leukocyte activation, leukocyte-mediated immunity, cytokine production, immune response-activating cell surface receptor signaling pathway, activation of the innate immune response, Th17 cell differentiation, cytokine–cytokine receptor interaction, and TNF signaling pathway, were significantly associated with TMPRSS2 expression (Figures 6 and 7). Considering the relationship between TMPRSS2 and the immune response, low TMPRSS2 expression in lung cancer patient tissues may lead to a decline in the immune function of patients with SARS-CoV-2 infection. Intriguingly, we observed that TMPRSS2 expression correlated with infiltrating levels of CD8+ T cells, B cells, CD4+ T cells, neutrophils, macrophages, and dendritic cells in lung cancer (Figure 8). Additionally, we found that TMPRSS2 was obviously associated with various gene set markers of different types of immune cells (Tables 1–3). According to the results of single-cell RNA-seq analysis, TMPRSS2 was expressed not only in colorectal epithelial cells but also in master cells, macrophages, B cells and T cells in colorectal cancer tissues [23]. This finding may be one of the reasons why lung cancer patients are more likely to be infected with this novel coronavirus.

To confirm the change in TMPRSS2 expression after SARS-CoV-2 infection, we utilized four GEO datasets. The expression of TMPRSS2 in three datasets, GSE33267, GSE47962, and GSE45042, was significantly reduced in response to SARS-CoV infection (Figure 11). We also investigated the effect of SARS-CoV-2 infection on TMPRSS2 expression in Vero E6 cells. Although there was no significant difference, TMPRSS2 expression exhibited a decreasing trend (Figure 11). In fact, in the GSE156544 dataset, there were only two samples of SARS-CoV-2 infection. Because SARS-CoV-2 shares high homology with SARS-CoV, the TMPRSS2 expression level may similarly be reduced with SARS-CoV-2 infection. The downregulated expression of TMPRSS2 caused by SARS-CoV-2 infection may aggravate a variety of symptoms. Lung cancer patients should take adequate preventive measures to avoid COVID-19 infection and continuously monitor cell metabolism- and immune-related indicators [43].

In summary, we systematically analyzed the clinical significance and molecular mechanism of TMPRSS2 in lung cancer. TMPRSS2 expression was significantly downregulated in lung cancer. Decreased TMPRSS2 related with a poor prognosis and was associated with immune cell infiltration in lung cancer. The DNA methylation level of the TMPRSS2 promoters showed marked increases in LUAD and LUSC, indicating a potential cellular mechanism of TMPRSS2 gene expression in lung cancer. More importantly, TMPRSS2 expression was significantly decreased during SARS-CoV infection. Based on these results, we identified and elucidated the important roles of TMPRSS2 in lung cancer and the underlying mechanisms associated with its immune infiltration. However, there are several limitations. First, we did not perform *in vitro* or *in vivo* experiments to validate the precise roles and molecular mechanisms of TMPRSS2 in lung cancer. Further studies are required to confirm the prognostic value and mechanism by which TMPRSS2 influences the oncogenesis of lung cancer. Second, the present study lacks clinical information on lung cancer combined with SARS-CoV-2 infection data. Third, several variants in TMPRSS2 have been recently identified to affect the structure, function and stability of TMPRSS2. These variants may affect susceptibility to SARS-CoV-2 infection and lung cancer which needs to be confirmed in further studies. Although the lung is the primary target organ for COVID-19, it is necessary to identify TMPRSS2 expression in different cell types of lung which may affect the variable susceptibility to SARS-CoV-2 infection.

## MATERIALS AND METHODS

### Oncomine database analysis

The Oncomine database (http://www.oncomine.org) was used to determine TMPRSS2 expression in lung cancer tissues and adjacent corresponding normal tissues [44–48]. The investigation was carried out based on the following criteria: *P* value, <0.01; fold change, < −2.5; and gene ranking, all.

### GEPIA2 database

We used GEPIA2 (http://gepia2.cancer-pku.cn/#index) to examine the mRNA expression level of TMPRSS2 in lung cancer and validate the correlation between TMPRSS2 and the expression levels of candidate genes [44–48].

### UALCAN database

UALCAN (http://ualcan.path.uab.edu/), an online database containing transcriptome data from a variety of human cancers, was used to investigate the expression level and DNA methylation level of TMPRSS2 for comparisons not only between lung cancer and normal tissues but also across multiple subgroups stratified by clinicopathological parameters, such as sex, tumor stage, tumor grade and race.

### TIMER database

The correlations between TMPRSS2 expression and the abundance of immune cell infiltrates in lung cancer datasets were analyzed using the TIMER database (https://cistrome.shinyapps.io/timer/) [44–48]. Correlations between TMPRSS2 expression and various gene marker sets of tumor-infiltrating immune cells were determined through a correlation module. The gene expression levels are represented as log2 TPM values.

### cBioPortal database

The cBioPortal database enables users to investigate genomic profiles, such as the genetic alterations, survival curves and correlations of TMPRSS2 in lung cancer.

### Kaplan–meier plotter analysis

The Kaplan–Meier plotter was applied to evaluate the prognostic value of TMPRSS2 in OS, FPS and PPS in lung cancer.

### PrognoScan database

We used the PrognoScan database (http://www.prognoscan.org/), a comprehensive and user-friendly database with clinical annotation, to assess the relationship between TMPRSS2 expression and prognostic information, including OS and RFS, in lung cancer patients. Cox *P* values and HRs with 95% confidence intervals were automatically calculated.

### STRING and GeneMANIA databases analyses

GeneMANIA was applied to construct a gene–gene interaction network for TMPRSS2 in terms of physical interactions, coexpression, predictions, colocalization, and genetic interaction, as well as to evaluate their functions [44–48]. In addition, STRING database was used to develop a PPI network.

### KEGG, GO and GSEA

KEGG and GO analyses were applied to examine the functions of TMPRSS2 in lung cancer. GO analysis was used to assess the biological processes (BP), molecular functions (MF) and cellular components (CC) related with TMPRSS2. We also applied GSEA to examine the potential mechanisms of TMPRSS2 in lung cancer. All of these analyses were performed using the R package ClusterProfiler [44–48].

### CIBERSORT estimation

We used the CIBERSORT algorithm to identify the fractions of immune cells based on bulk samples from the LUAD and LUSC cohorts. The associations between TMPRSS2 expression and immune cell infiltration levels were evaluated using Spearman’s correlation test.

### IHC analysis

The TMPRSS2 protein expression in lung cancer and normal lung tissues from the HPA (Human Protein Atlas) database (https://www.proteinatlas.org/) were investigated by IHC staining.

### SurvivalMeth

The SurvivalMeth online database was used to assess the DNA methylation of the TMPRSS2 gene and the influence of DNA methylation of TMPRSS2 on prognosis in LUAD and LUSC [44–48].

### Cox regression analysis

Univariate and multivariate Cox regression analyses were carried out to evaluate the association between TMPRSS2 expression and OS of lung cancer patients using the TCGA database. The forest was generated to show the *P* value, HR and 95% CI of each clinicopathologic parameter through the R package “forestplot”.

### Construction and evaluation of a nomogram

Based on clinical characteristics, we generated a nomogram to predict the probability of OS using the R package “rms” (https://www.rdocumentation.org/packages/rms). The C-index was calculated to estimate the predictive accuracy. Calibration curves were plotted to compare the predicted OS with actual OS rates.

### Open Targets platform

The Open Targets platform (http://www.targetvalidation.org) was used to identify the associations of TMPRSS2 and human diseases.

### Data availability

The data used to support the findings of this study are available from the corresponding author upon request.

## Supplementary Materials

Supplementary Figures
