# Supplementary material for: Decreased TMPRSS2 expression by SARS-CoV-2 predicts the poor prognosis of lung cancer patients through metabolic pathways and immune infiltration
Source: Aging (Albany NY). 2022 Jan 11;14(1):73–108. doi: 10.18632/aging.203823 (PMC8791221; doi:10.18632/aging.203823)
Supplement: Supplementary Figures [file aging-14-203823-s001.pdf]

SUPPLEMENTARY FIGURES

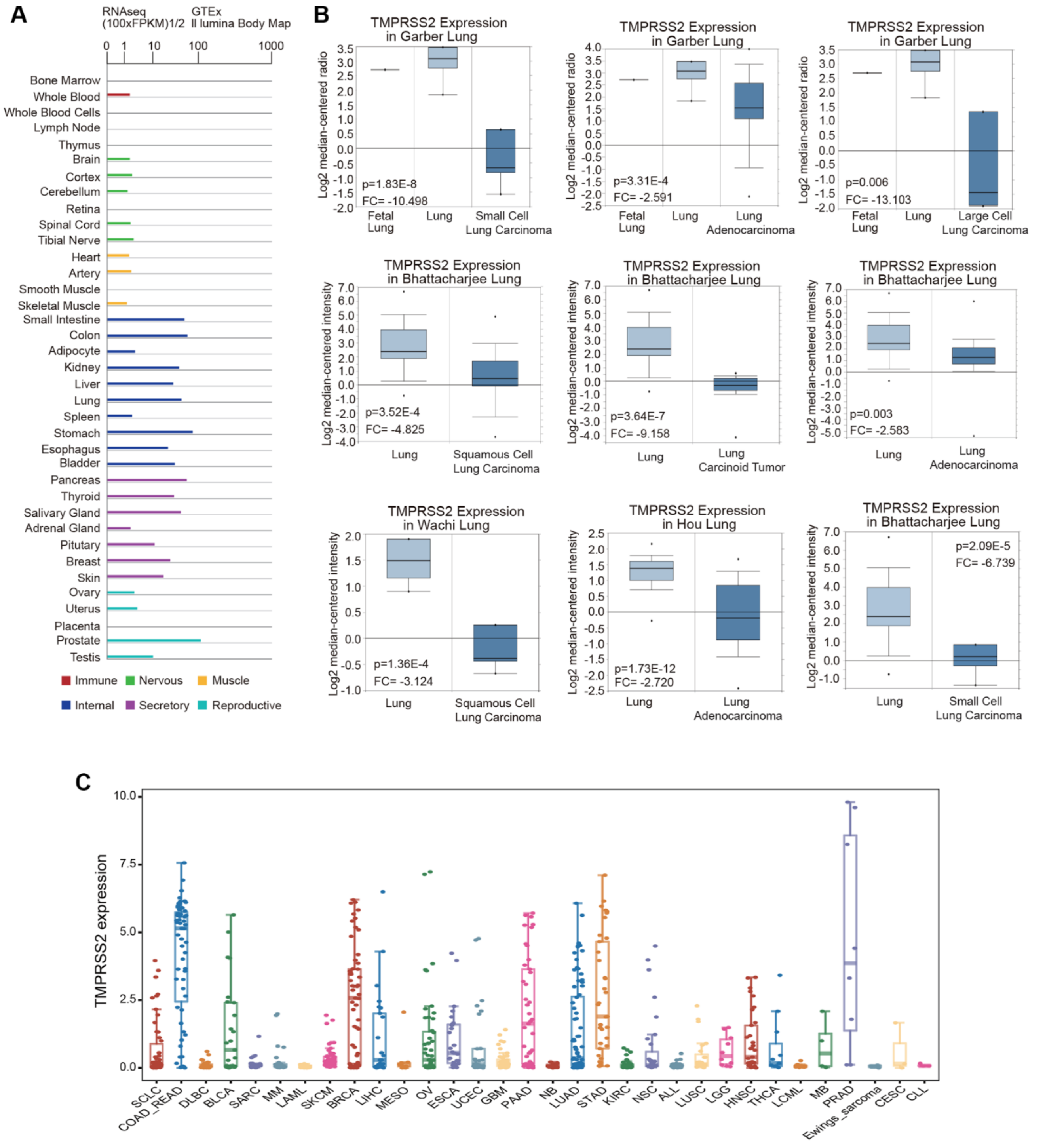

Supplementary Figure 1. (**A**) TMPRSS2 expression in different organs and tissues. (**B**) TMPRSS2 expression in different types of lung cancer patients and normal individuals from the Oncomine database. (**C**) TMPRSS2 expression in different types of cancer cells using the CCLE database.

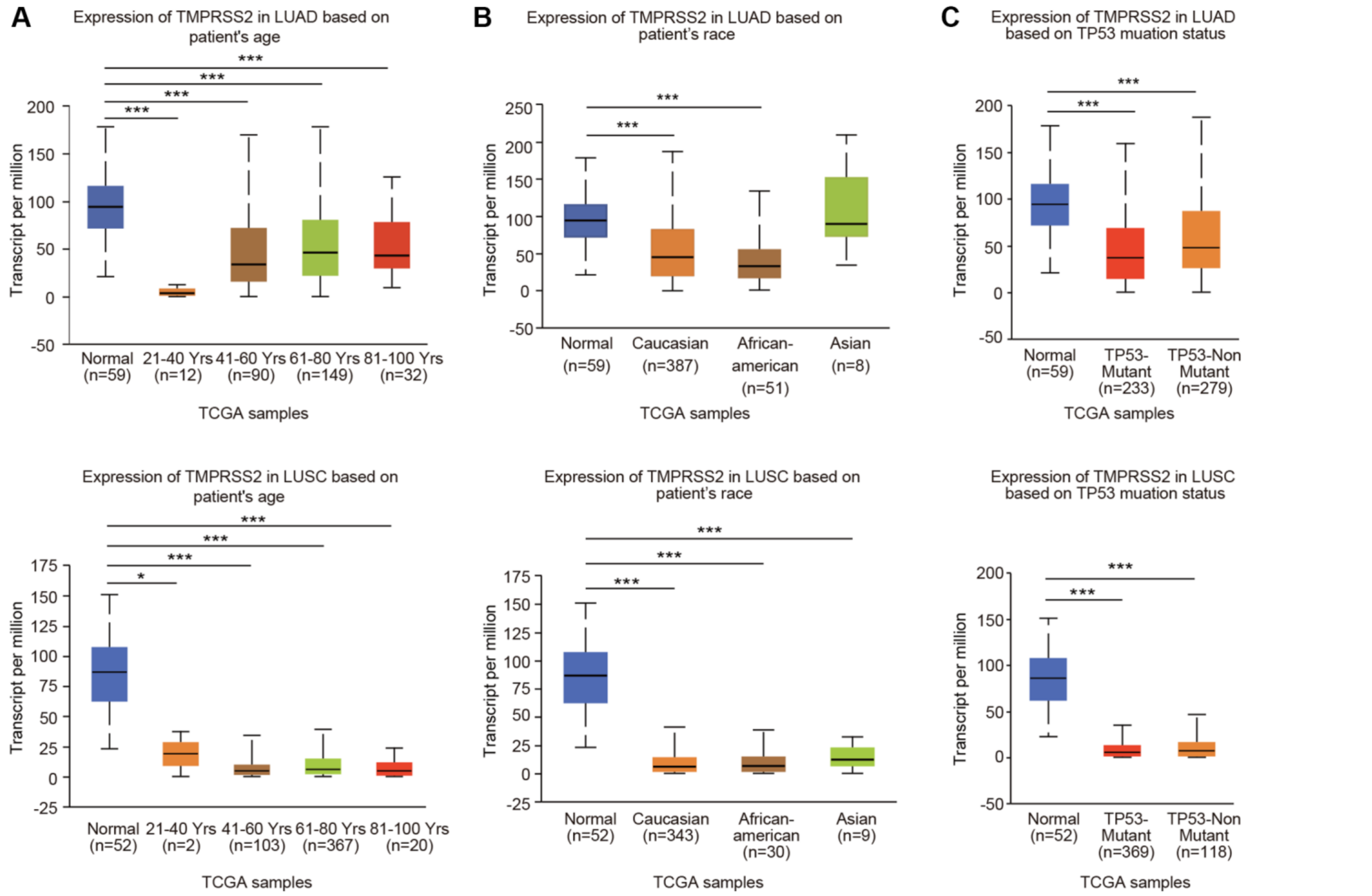

Supplementary Figure 2. TMPRSS2 expression was assessed in (**A**) patients with different ages, (**B**) patients with different races, (**C**) patients with different TP53 statuses from the UALCAN database.

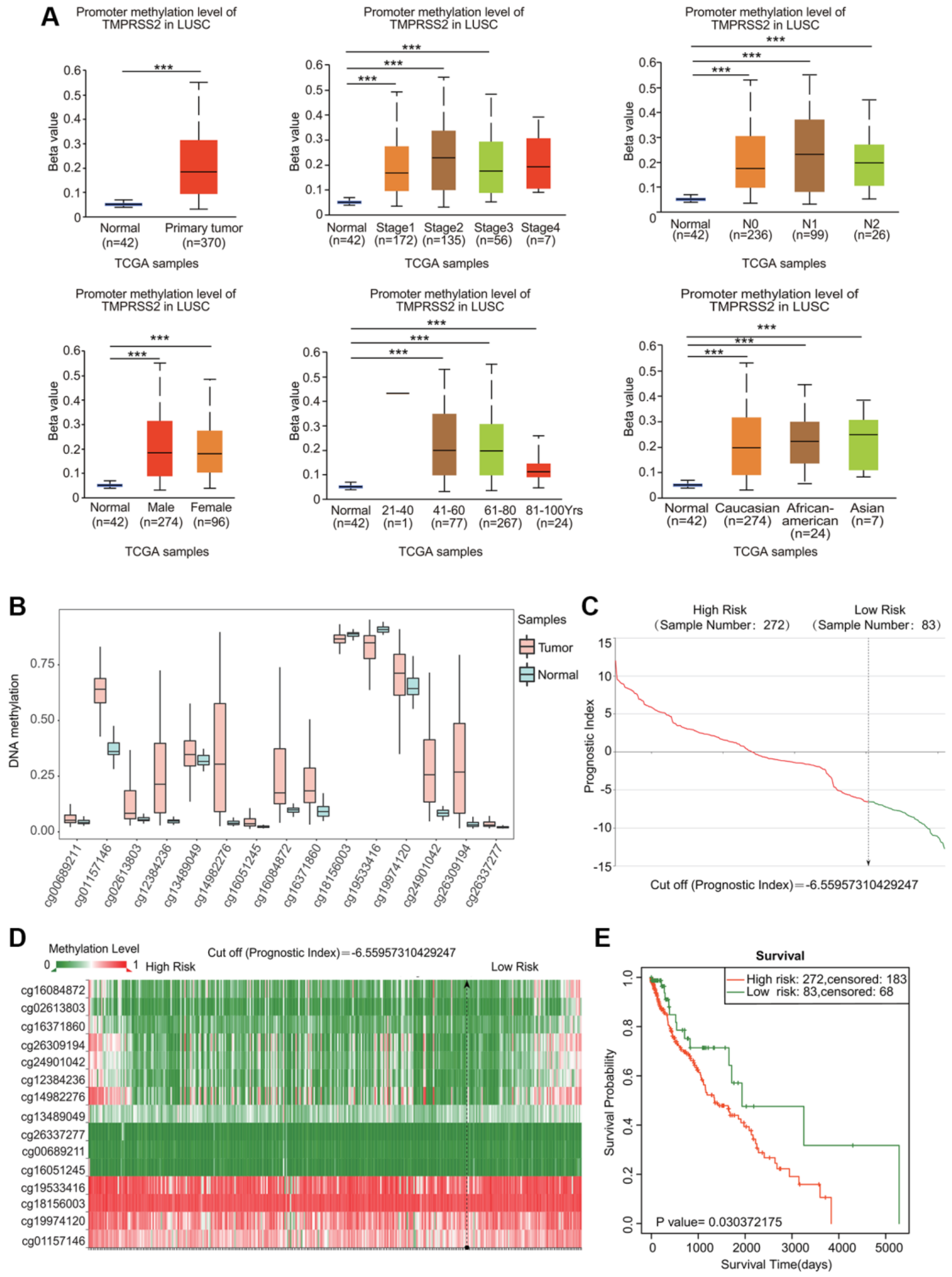

Supplementary Figure 3. DNA methylation of TMPRSS2 in LUSC. (**A**) Association of DNA methylation of TMPRSS2 with clinicopathological parameters of LUSC. (**B**) Methylation levels of TMPRSS2 in LUSC according to the SurvivalMeth database. (**C**) The distribution of prognostic index in LUSC. (**D**) The heatmap of DNA methylation of TMPRSS2 in LUSC. (**E**) The prognostic potential of DNA methylation of TMPRSS2 in LUSC based on the SurvivalMeth database.

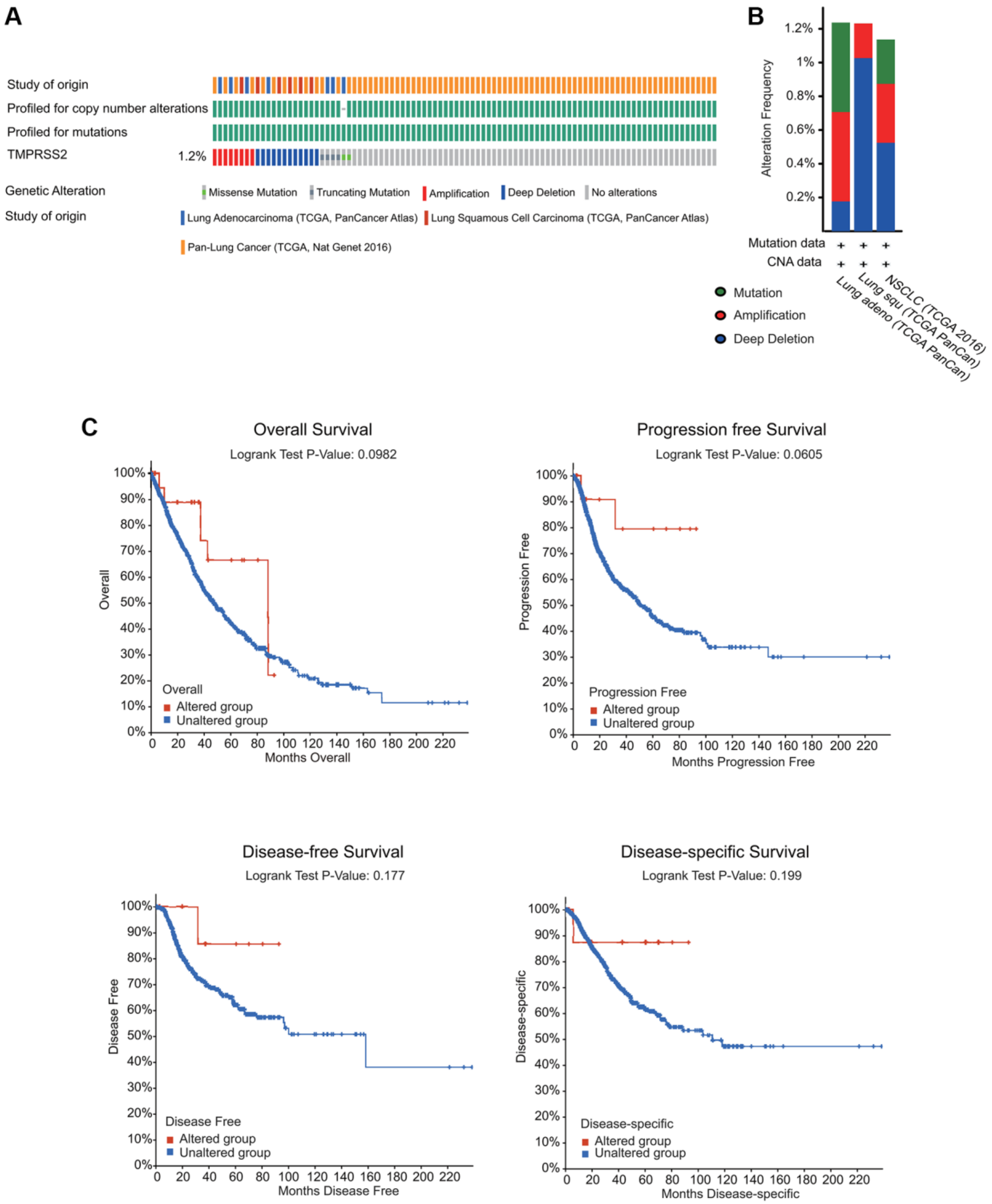

Supplementary Figure 4. Alteration frequency of TMPRSS2. (**A**) OncoPrint visual summary of alterations on a query of TMPRSS2 from the cBioPortal database. (**B**) Summary of TMPRSS2 genetic alterations in lung cancer. (**C**) Kaplan-Meier plots comparing OS, PFS, DFS and DSS in cases with or without TMPRSS2 gene alterations from the cBioPortal database.

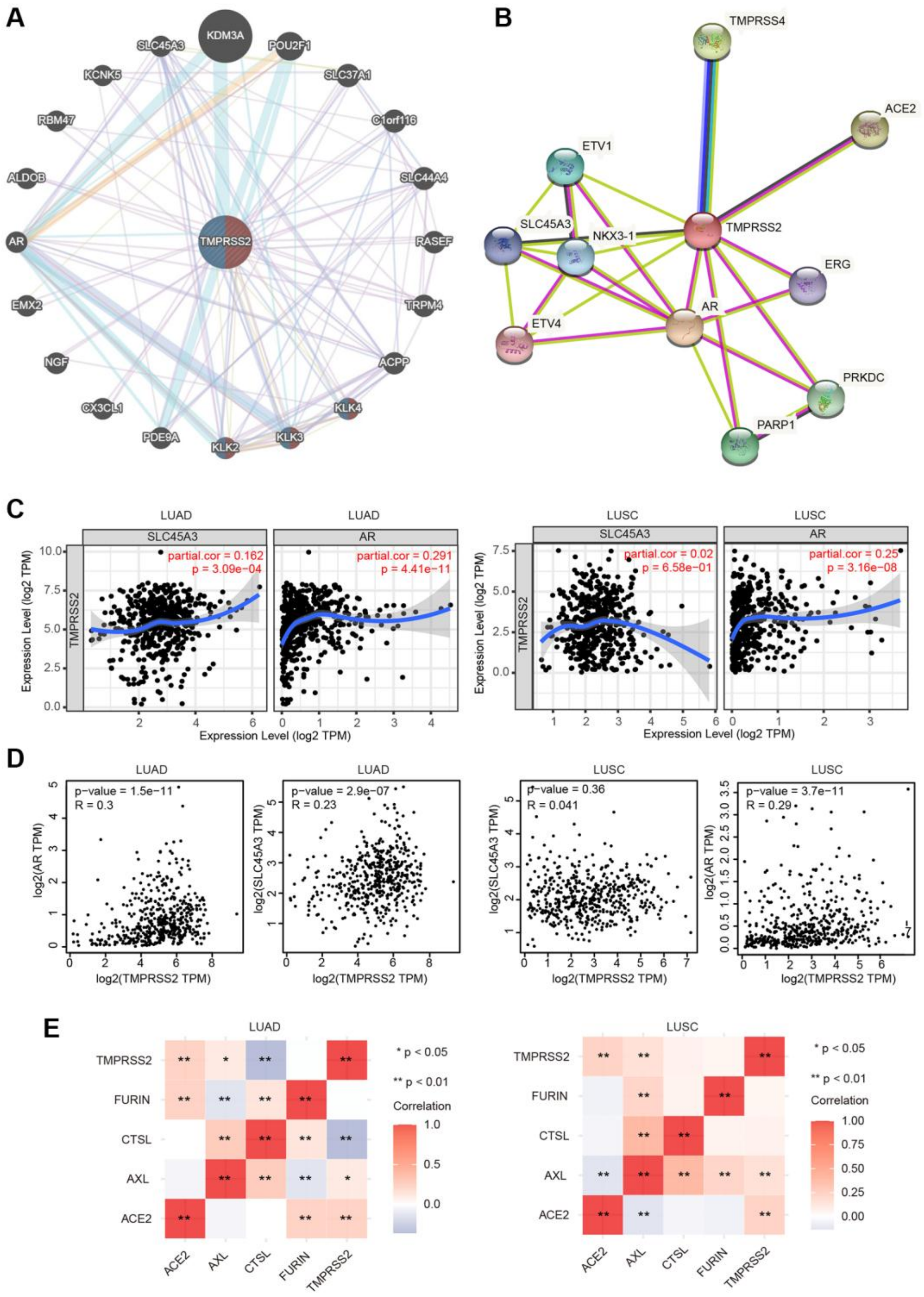

Supplementary Figure 5. Analysis of neighboring gene networks in lung cancer. (**A**) The gene-gene interaction network of TMPRSS2 was constructed using GeneMANIA. (**B**) The PPI network of TMPRSS2 was constructed using STRING. (**C**, **D**) Scatterplots of the correlations between TMPRSS2 expression and SLC45A3 and AR expression in lung cancer using the TIMER and GEPIA databases, respectively. (**E**) Heatmap of correlations between TMPRSS2 expression and other targets of COVID-19 therapy in LUAD and LUSC.

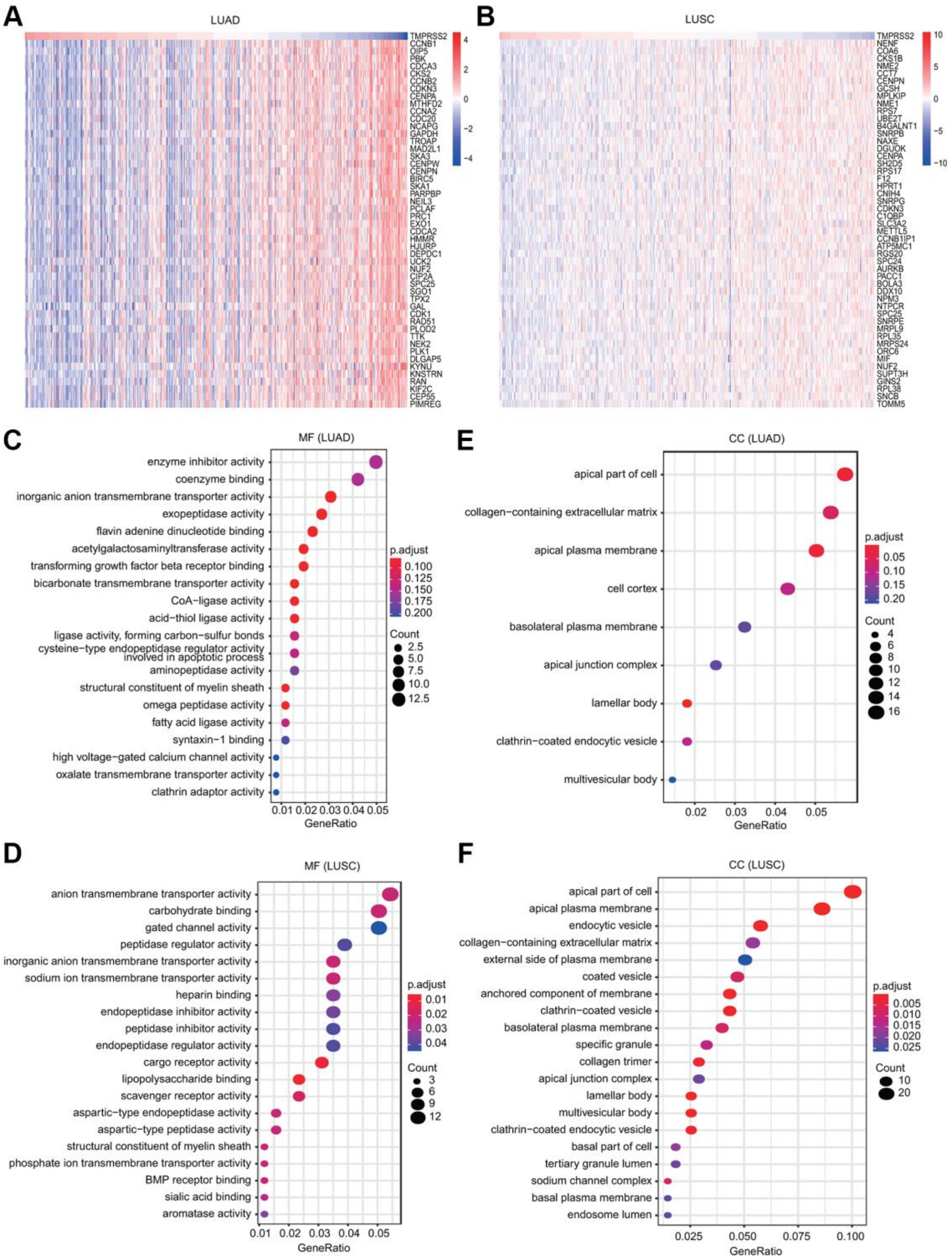

Supplementary Figure 6. GO and KEGG analyses for TMPRSS2 in lung cancer. (**A**, **B**) Heat maps showing the top 50 genes that were negatively associated with TMPRSS2 in LUAD and LUSC, respectively. (**C**, **D**) Top 20 enrichment terms in the MF category in LUAD and LUSC, respectively. (**E**, **F**) Top 20 enrichment terms in the CC category in LUAD and LUSC, respectively.

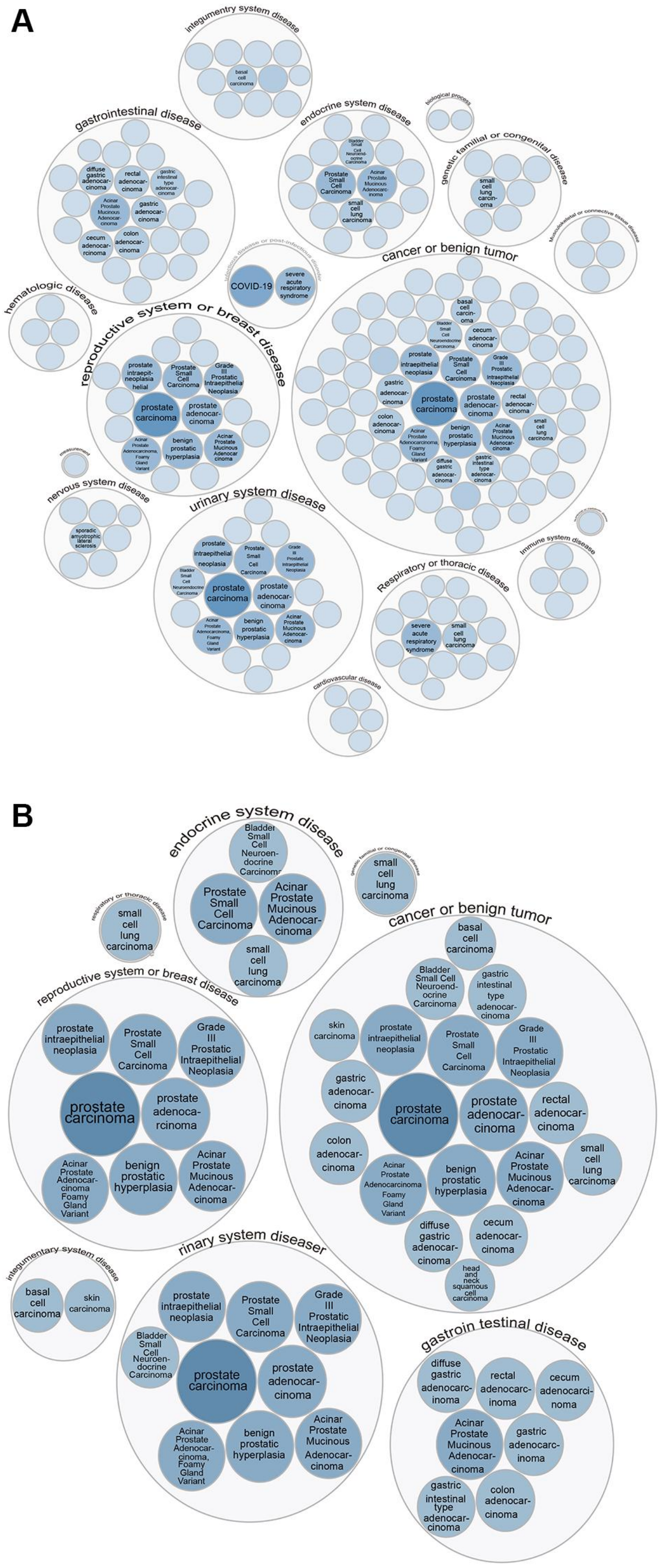

Supplementary Figure 7. (**A**) TMPRSS2 expression was related with various human diseases. (**B**) TMPRSS2 expression was related with multiple cancerous diseases using the Open Targets platform.

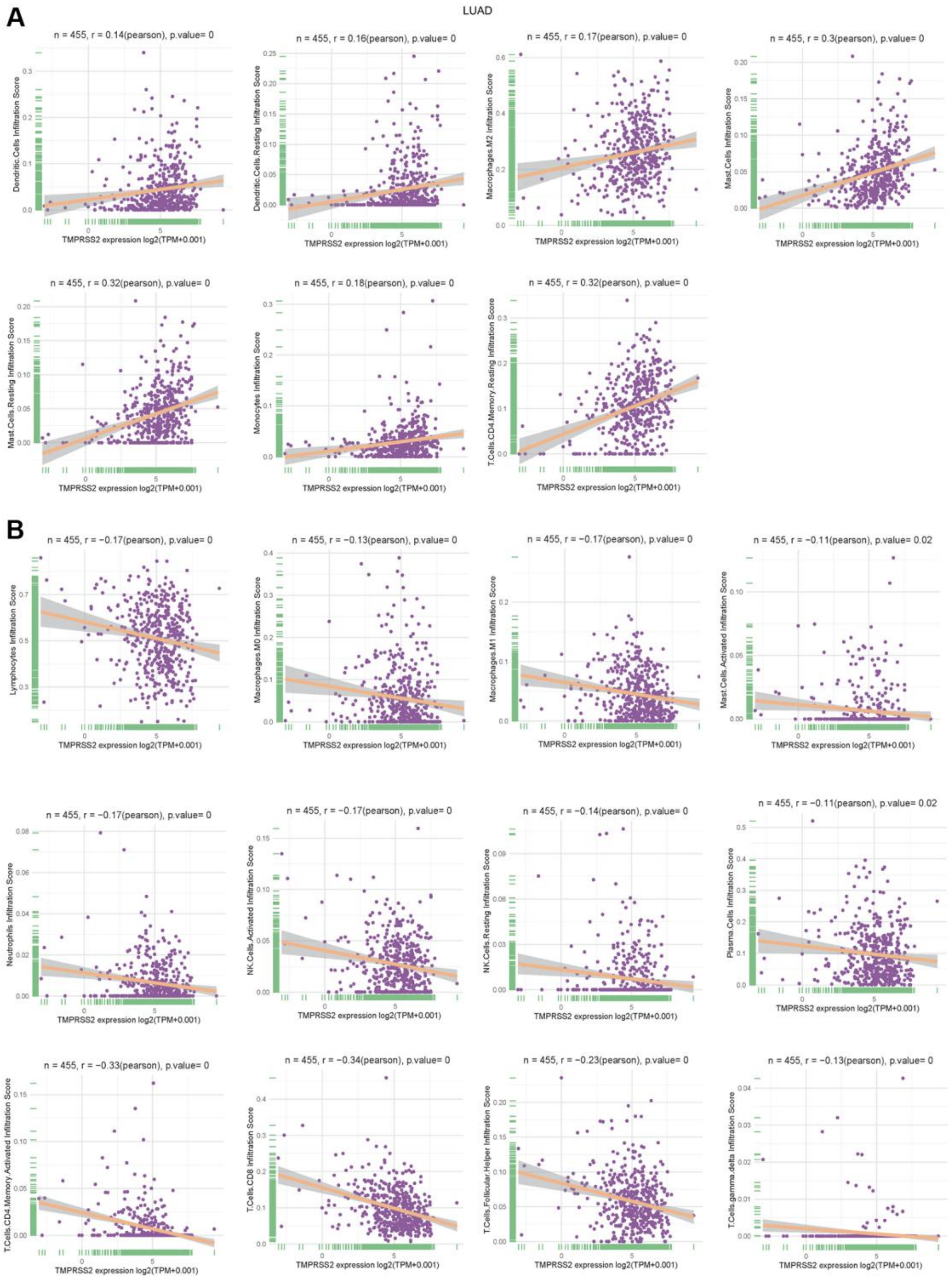

Supplementary Figure 8. (**A**, **B**) TMPRSS2 expression was positively or negatively correlated with the infiltration of different immune cells in LUAD according to the CIBERSORT algorithm.

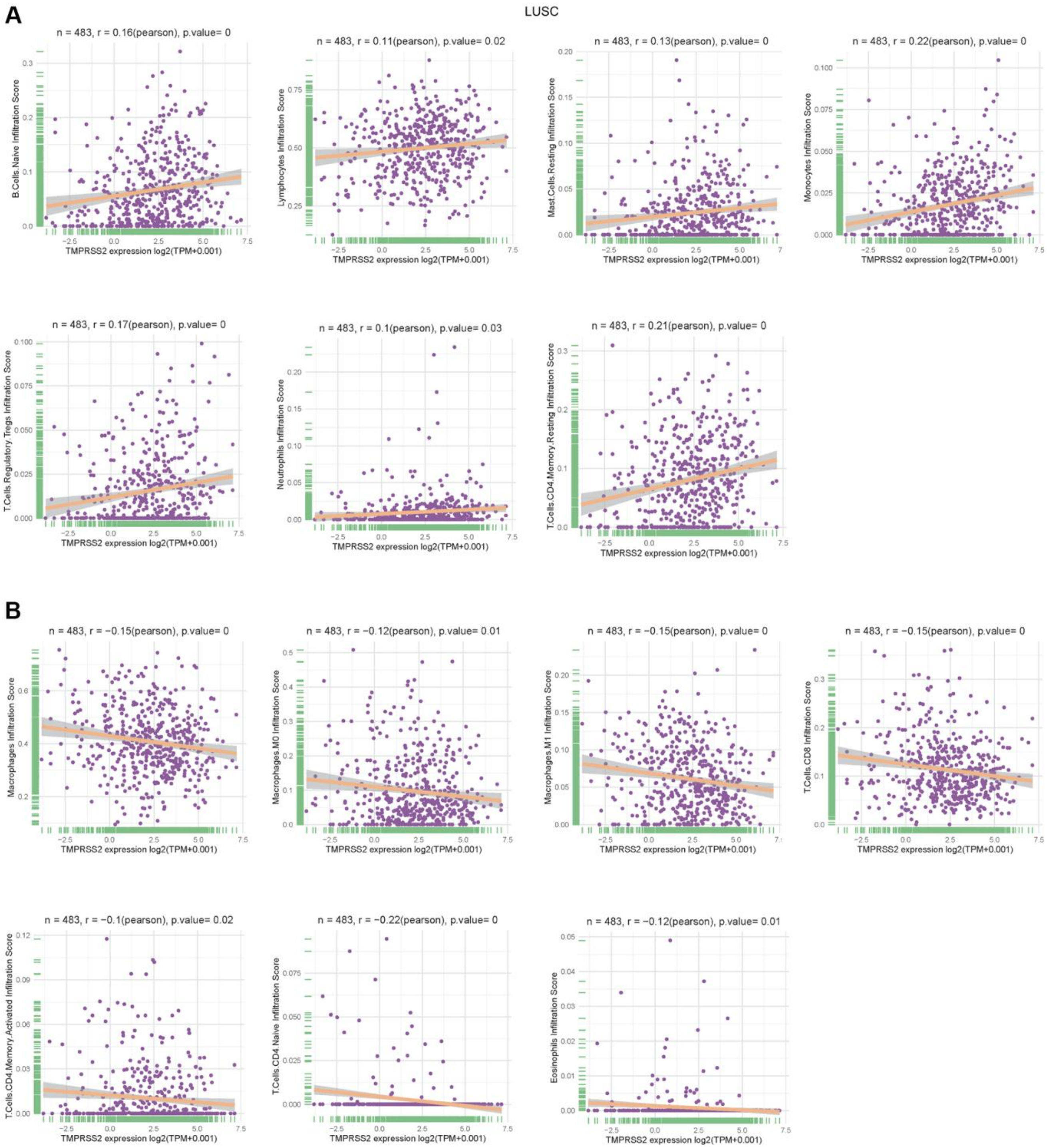

Supplementary Figure 9. (**A**, **B**) TMPRSS2 expression was positively or negatively correlated with the infiltration of different immune cells in LUSC according to the CIBERSORT algorithm.

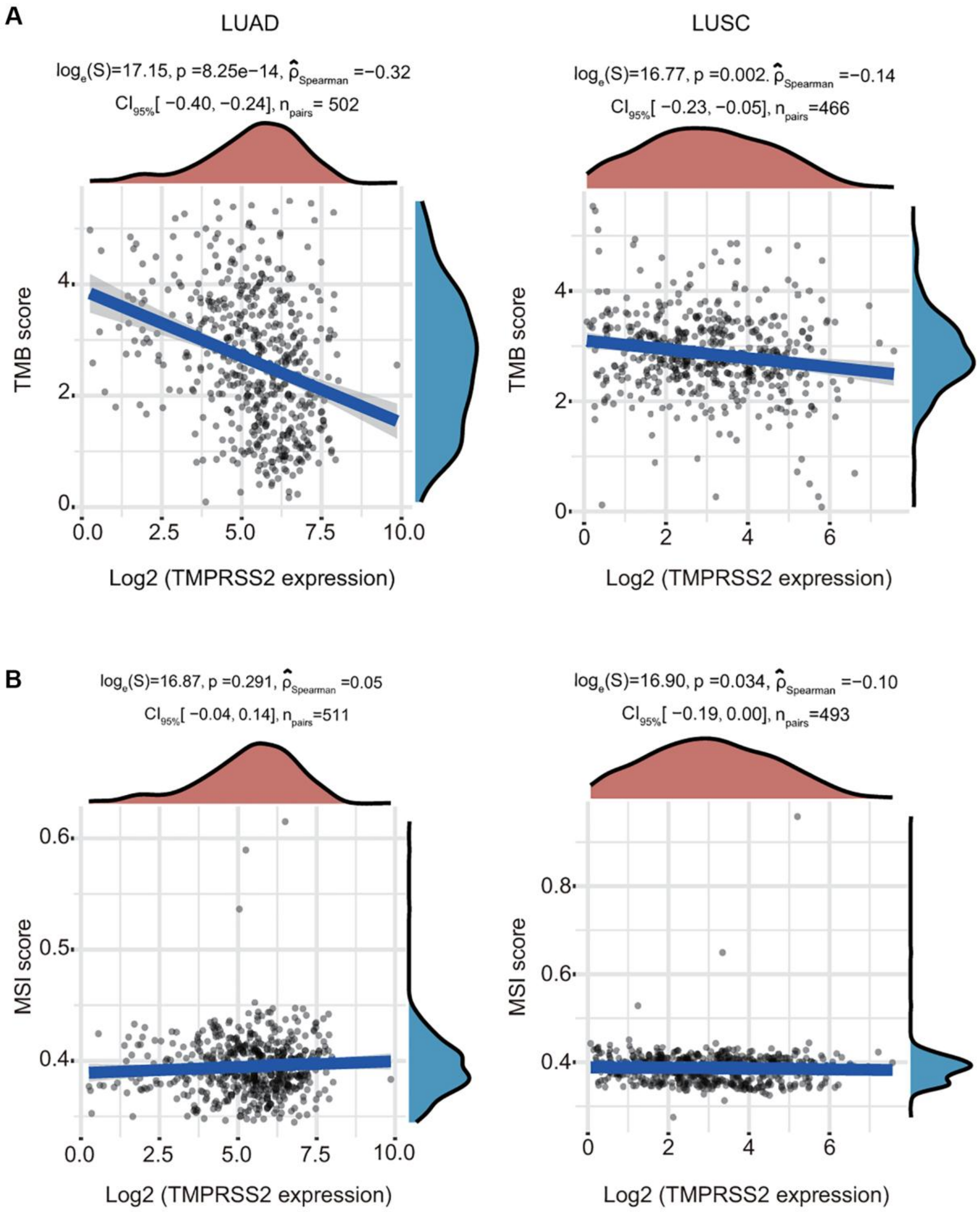

Supplementary Figure 10. (**A**, **B**) Correlations between TMPRSS2 expression and TMB and MSI in LUAD and LUSC.

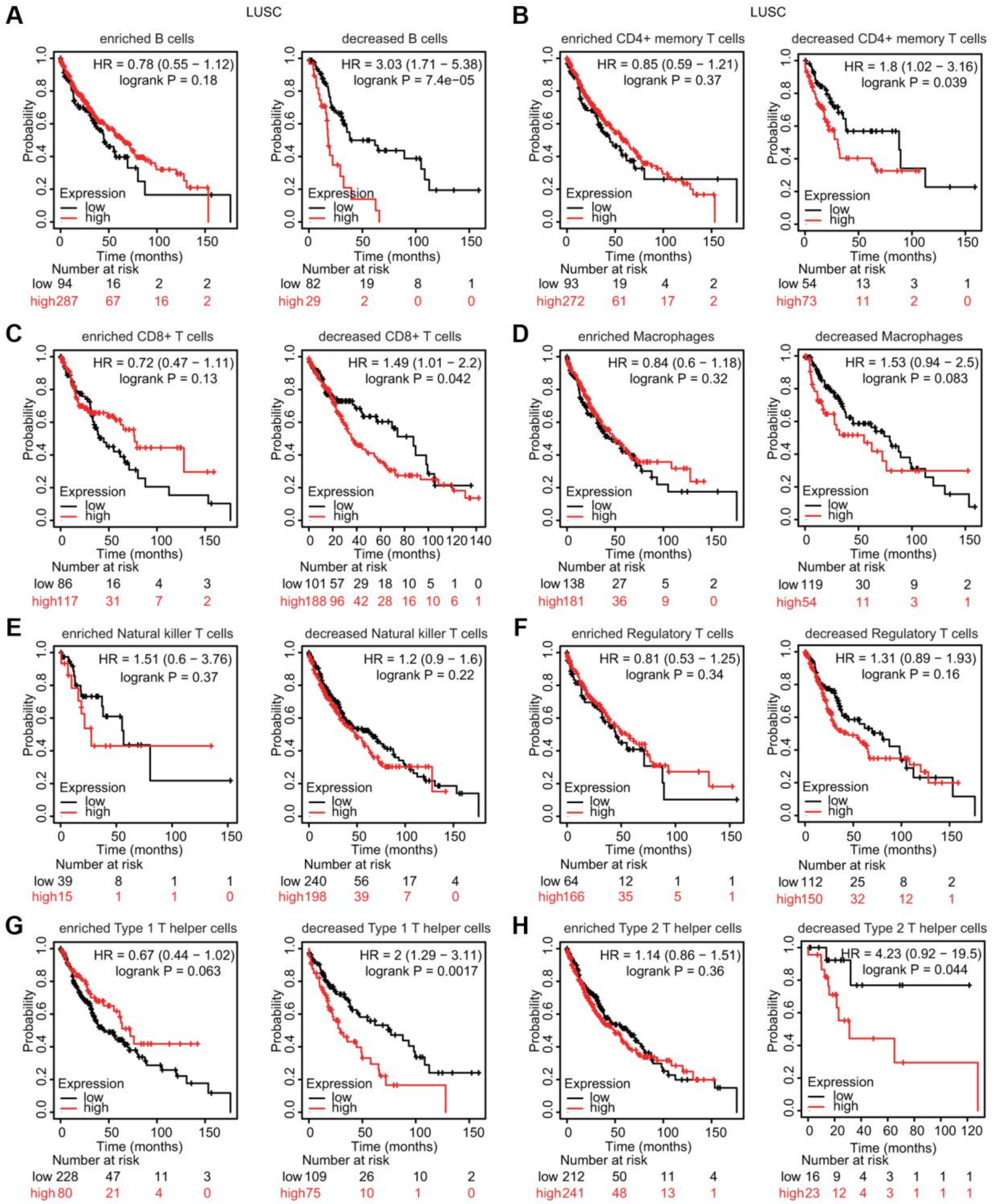

Supplementary Figure 11. (**A**–**H**) Correlations between TMPRSS2 expression and OS in different immune cell subgroups of LUSC patients were examined using the Kaplan-Meier plotter database.
